# Ophthalmic Nanoemulsions: From Composition to Technological Processes and Quality Control

**DOI:** 10.1021/acs.molpharmaceut.1c00650

**Published:** 2021-09-17

**Authors:** Agnieszka Gawin-Mikołajewicz, Karol P. Nartowski, Aleksandra J. Dyba, Anna M. Gołkowska, Katarzyna Malec, Bożena Karolewicz

**Affiliations:** Department of Drug Form Technology, Wroclaw Medical University, Borowska 211 A, 50-556 Wroclaw, Poland

**Keywords:** nanoemulsion, ophthalmic nanoemulsion, ocular drug delivery, emulsification, low-energy methods, high-energy methods

## Abstract

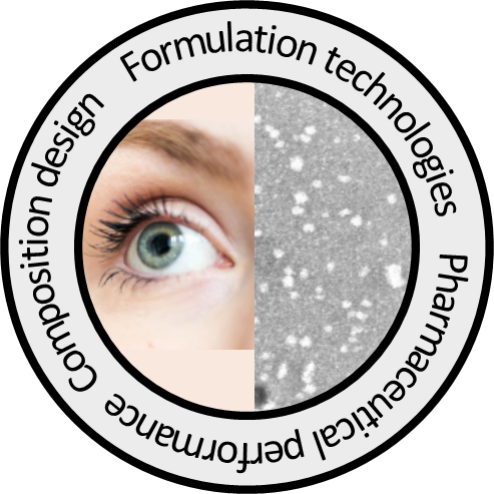

Nanoemulsions are considered as the most promising solution to improve the delivery of ophthalmic drugs. The design of ophthalmic nanoemulsions requires an extensive understanding of pharmaceutical as well as technological aspects related to the selection of excipients and formulation processes. This Review aims at providing the readers with a comprehensive summary of possible compositions of nanoemulsions, methods for their formulation (both laboratory and industrial), and differences between technological approaches, along with an extensive outline of the research methods enabling the confirmation of *in vitro* properties, pharmaceutical performance, and biological activity of the obtained product. The composition of the formulation has a major influence on the properties of the final product obtained with low-energy emulsification methods. Increasing interest in high-energy emulsification methods is a consequence of their scalability important from the industrial perspective. Considering the high-energy emulsification methods, both the composition and conditions of the process (e.g., device power level, pressure, temperature, homogenization time, or number of cycles) are important for the properties and stability of nanoemulsions. It is advisible to determine the effect of each parameter on the quality of the product to establish the optimal process parameters’ range which, in turn, results in a more reproducible and efficient production.

## 1 Introduction

Ophthalmic therapy is largely based on topical administration of aqueous active pharmaceutical ingredients (API) solutions which are eliminated shortly after instillation by the nasolacrimal drainage system.^1,2^ The short residence time of the conventional ocular formulations (i.e., eye drops) in the conjunctival sac is limiting drug penetration into the deeper layers of the eye to less than 5% of the administered dose,^3^ while other reports indicate that the effectively absorbed ocular dose is smaller than 1% of the instilled dose.^4^ The reason for that is rapid drug elimination due to blinking and lachrymation, which is amplified by the administration of nonphysiological concentrations of substances to the eye (e.g., in the form of eye drops or suspensions).^5^ Furthermore, factors such as drug binding to tear fluid proteins, drug metabolism facilitated by enzymes present in the tear fluid, and poor corneal permeability have been found to be responsible for low bioavailability of the drugs after their topical administration to the eye.^6^ Despite the above-mentioned challenges, a significant number of commercial ophthalmic preparations are available exclusively in the form of droplets and suspensions. The interest in further development of topically applied ocular formulations is a consequence of its convenient, noninvasive way of drug application, patients’ compliance, and low production costs.^5,7^ Apart from the formulation of conventional topical drug dosage forms for ophthalmic diseases (i.e., solutions, emulsions, suspensions, gels, and ointments), there is an increasing interest in the development of novel, advanced ocular carriers including nanomicelles, nanoemulsions, liposomes, dendrimers, implants, nanosuspensions, or *in situ* thermosensitive gels.^7,8^ Even though commercially available nanocarriers are still rare, nanotechnology-based formulations are currently considered as the most promising systems enabling the improvement of API delivery to the eye.^9^ Out of the 11 nanotechnology-based ophthalmic formulations approved by the FDA by the end of 2020, there are 3 nanoemulsions, 3 nanosuspensions, 3 biodegradable and nonbiodegradable implants, 1 composed with liposomes, and 1 with nanoparticles.^10^ Currently marketed ophthalmic nanosize drug delivery systems include micelles (Cequa), liposomes (VISUDYNE, Lacrisek, Artelac Rebalance), and nanoemulsions (Restasis, Cyclokat, Ikervis, Durezol, Xelpros, Systane Complete).^7,10−13^ Several nanoemulsions were also in advanced clinical evaluation.

These include Vekacia (cationic nanoemulsion containing 0.05% or 0.1% cyclosporin A) in the treatment of vernal keratoconjunctivitis and the Catioprost (nanoemulsion containing 0.005% latanoprost) in the treatment of glaucoma.^11^ Among the marketed nanoemulsions registered for the treatment of dry eye syndrom, keratitis, and glaucoma formulations that do not contain any API can also be found. Ocular nanoemulsions provide large surface contact of the carrier with the eyeball that can result in improved corneal permeability, increased bioavailability, and efficacy. The presence of surface active ingredients in nanoemulsions enables enhanced mixing of nanosize droplets with the precorneal constituents and, as a consequence, a greater dispersion of the drug over the cornea. This results in a prolonged contact time of the drug with the corneal epithelium and a rapid onset of action.

During development of other nanosized systems for ocular drug delivery (e.g., nanosuspensions, nanoparticles, implants), one should consider the nanoparticles’ aggregation during storage and in contact with tear fluid, their toxicity, biodistribution, elimination, or invasive administration. Inorganic and metal nanoparticles are usually nonbiodegradable, and their elimination from the eye structures is long and cell cycle-dependent. Biodegradable, nontoxic polymer nanoparticles provide a versatile platform for tailored ocular delivery of various API. In contrast to soft colloidal nanomaterials (nanoemulsions, liposomes), solid nanomaterials applied to the eyeball may aggregate either in contact with tear fluid or after corneal barrier permeation affecting their *in vivo* performance. This imposes the requirement for the use of advanced biological models in order to fully understand the *in vivo* performance of these formulations. Furthermore, the insufficient regulatory framework and the potential complexity of scaling-up processes may increase the development costs of new formulations.^10,11^

Nanoemulsions are transparent, kinetically stable formulations with inner-phase droplets typically in the range of 20–200 nm (some authors expand the upper limit of the dispersed particles size to 500 nm).^14,15^ Ophthalmic o/w nanoemulsions are composed of a dispersed phase (oil), a continuous phase (water), and a carefully selected composition of surfactants and cosurfactants enabling the reduction of the surface tension at the interphase of two immiscible phases of the nanoemulsion.^14^

Nanoemulsions applied to the eye, because of the specificity of the application site, require the addition of auxiliary substances such as preservatives, tonicity modifiers, buffering agents, viscosity enhancers, antioxidants, and API solubilizers. Their role is to preserve and increase physical stability of the formulation as well as to improve its physicochemical properties (e.g., decrease droplet size or increase the colloidal stability).^16^ The prolonged precorneal retention time and high penetration capacity of nanoemulsions result in enhanced ocular bioavailability.^17^ The surfactants and cosurfactants present in nanoemulsion formulations act as penetration enhancers facilitating corneal transport of the drug. They remove the mucus layer and disrupt tight junctional complexes enabling increased API penetration into the deeper layers of the eye. Furthermore, ocular absorption of the drug from nanoemulsions is enhanced by the submicron size of the particles transported via endocytosis through the corneal epithelial cells. Effective administration of topical medications requires correct eye drops application, the correct number of administrations per day (up to 4 doses per day), and the correct interval between several doses or other ophthalmic medications. In practice, compliance with medical recommendations for various diseases (e.g., glaucoma, corneal infections of various type, dry eye syndrome, or immune-mediated inflammatory anterior ocular diseases) regarding treatment with topical medications is poor, and studies suggest that in glaucoma, for example, less than half of the patients are able to maintain the recommended dosing regimens.^18−23^ Increased ocular absorption of the drug from nanoemulsions, as compared with conventional eye drops, may reduce the number of doses and ultimately simplify a dosing scheme, resulting in better patients' compliance.^17,18^ Dissolution of hydrophobic API in the lipid phase of a nanoemulsion provides reproducible dose administration as compared with ophthalmic suspensions, gels, and ointments.^24−27^ For example, the o/w nanoemulsion with 0.4% concentration of brinzolamide confirmed enhanced penetration into the corneal tissue and higher therapeutic efficacy *in vivo* in comparison with the commercially available 1% brinzolamide suspension.^28^ Similarly, increased therapeutic efficacy and greater drug concentration in aqueous humor (as compared to commercial products) were reported for nanoemulsions containing dorzolamide hydrochloride,^29^ acetazolamide,^30^ moxifloxacin,^31^ terbinafine hydrochloride,^32^ and tacrolimus.^33^

These results suggest that the therapeutic efficacy of ophthalmic drugs administered as nanoemulsions may be achieved using a simplified and less frequent dosing regimens as compared with conventional products.

**Figure 1:**
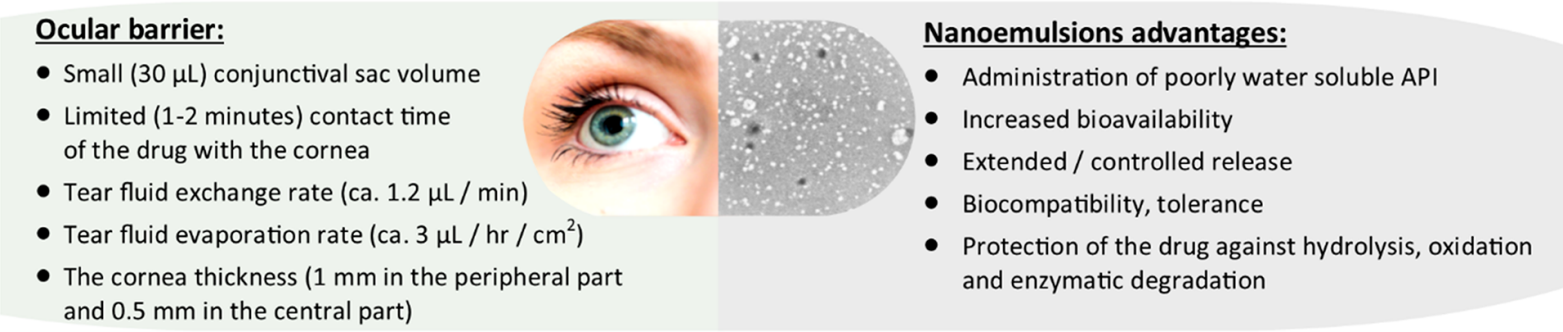
Characteristics of the ocular barrier and advantages of the ocular nanoemulsion formulations.

Despite the confirmed therapeutic benefit and increasing interest in the development of ophthalmic nanoemulsions, formulation of these novel drug delivery systems poses a significant challenge because of their multicomponent nature, complex chemistry, and extensive optimization required to propose robust and stable formulations. Furthermore, laboratory-scale methods of nanoemulsions synthesis differ significantly from industrial scale manufacturing approaches (e.g., the use of large-scale homogenizers) which makes them difficult to directly transfer to an industrial setup. This Review aims at providing the readers with a comprehensive summary of the possible compositions of nanoemulsions, methods of their formulation (including both laboratory- and industrial-scale methods), differences between technological approaches, as well as an extensive outline of the research methods enabling to confirm their *in vitro* properties, pharmaceutical performance, and biological activity. In contrast to other excellent reviews on ocular nanoemulsions^17^ and nanocarriers,^7,34^ this work comes from a technological perspective on formulation of ophthalmic nanoemulsions. The properties of selected oils, HLB values of surfactants, formulation optimization protocols, descriptions and comparison of technological approaches as well as critical parameters affecting successful formulation are the information frequently sought after by pharmaceutical technologists and newcomers. In this manuscript we provide the examples of available technological methods in a tabular overview as well as research methods, which might help navigate through the increasing number of reports on pharmaceutical nanoemulsions. As an extensive insight into physiological aspects of the ocular barrier^1,2,6^ and the ocular administration of nanoemulsions has recently been revised^17^ the Introduction and Figure 1 only briefly summarize these aspects.

## 2 Composition of the Nanoemulsions Administered to the Eye

**Figure 2:**
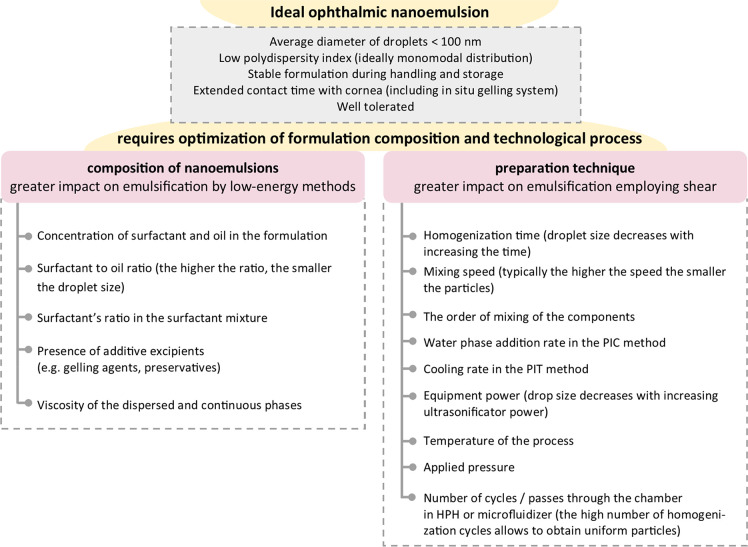
Optimization of the composition and technological processes required for formulation of an ideal ophthalmic nanoemulsion.

Ophthalmic o/w nanoemulsions can generally be considered as dispersions of oily droplets in an aqueous environment. Therefore, these formulations require careful selection of the composition of both the oily phase (i.e., the use of nontoxic, nonirritating, pharmaceutically approved oils) as well as the composition of the aqueous medium (Figure 2).

Similarly to the ophthalmic drops the need for isotonicity of the ocular nanoemulsions, the desired pH, a certain buffering capacity, the addition of preservatives (antimicrobial agents), viscosity modifiers, and antioxidants calls for careful consideration. Furthermore, in order to obtain nanosize uniform droplets of oils, an extensive optimization of the composition as well as the concentration of surfactants and cosurfactants in the formulation are required. The complexity of ophthalmic nanoemulsion compositions demands comprehensive knowledge and experience in pharmaceutical formulations so stable products of pharmaceutical quality could be designed. In this section we provide detailed characteristics of the most frequently used components of ophthalmic nanoemulsions including oils, emulsifiers, surfactants, and cosurfactants as well as additives used to modify their pharmaceutical properties (e.g., tonicity, viscosity, or pH of the formulation, Table 1).

**Table 1:** Components Used for Formulation of Ocular Nanoemulsions^9,17^^a^

component	examples
oil/lipid phase	castor oil, coconut oil, corn oil, evening primrose oil, linseed oil, mineral oil, olive oil, peanut oil, soybean oil, Capmul MCM, Capryol 90, Dermol M5, DOTAP, Estasan, ethyl oleate, Eutanol G, Epikuron 200, isopropyl myristate, Labrasol, Lipoid S75, Lipoid E80, Lipoid S100, MCT, Miglyol 812, oleic acid, Phospholipon 90H, triacetin, Transcutol, vitamin E
emulsifier/surfactant	castor oil derivatives, natural lecithins of plant or animal origin, phospholipids, polysorbates, stearylamine, Brij 35, Kolliphor RH60, Miranol C2M conc NP, Poloxamer 188, Poloxamer 407, Span 20, Span 40, Span 80, Soluphor P, Tween 20, Tween 40, Tween 80, Tyloxapol, vitamin E-TPGS
cosurfactant	Kolliphor EL, Kolliphor RH40, ethanol, glycerin, PEG 300, PEG 400, propylene glycol, polyene glycol, poloxamers, Miranol C2M conc NP, Soluphor P, triacetin, Transcutol P
tonicity modifiers	dextrose, glycerol, mannitol, propylene glycol, sorbitol, xylitol
additives	DOPE, DOTAP, lower alcohols (e.g., ethanol), propylene glycol, 1,3-butylene glycol, sugars such as glucose, sucrose, fructose, maltose, cetylpyridinium chloride, benzalkonium chloride, benzethonium chloride, cetrimide, cetalkonium chloride, stearylamine, oleylamine, poly(ethylenimine), poly(l-lysine)
antioxidant	ascorbic acid, tocopherol

a Brij 35 - polyoxyethylene glycol dodecyl ether, Capmul MCM - 60% of medium-chain monoglycerides and 40% of diglycerides derived from caprylic acid (83%) and capric acid (17%), Capryol 90 - propylene glycol monocaprylate, Kolliphor RH40 - macrogolglycerol hydroxystearate, Kolliphor RH60 - polyoxyl 60 hydrogenated castor oil, Kolliphor EL - macrogolglycerol ricinoleate, Dermol M5 - caprylic/capric triglyceride, DOPE - 1,2-di(9Z-octadecenoyl)-*sn*-glycero-3-phosphoethanolamine, DOTAP - 1,2-dioleoyl-3-trimethylammonium-propane, Epikuron 200 - soybean lecithin with phosphatidylcholine content >93%, Estasan (caprylic-capric-triglyceride), Eutanol G - 2-octyldodecanol, Labrasol - caprylocaproyl macrogolglyceride, Lipoid E80 - egg phospholipids with 80% phosphatidylcholine content, Lipoid S75 - soybean lecithin with 70% phosphatidylcholine content, Lipoid S100 - soybean phospholipids, phosphatidylcholine content: ≥ 94%, MCT - medium-chain triglycerides, Miglyol 812 - triglyceride ester of saturated coconut/palm kernel oil derived caprylic and capric fatty acids and plant derived glycerol, Miranol C2M conc NP - disodium cocoamphodiacetate, Phospholipon 90H - hydrogenated soybean phospholipids, phosphatidylcholine content: ≥ 90%, Polysorbate/Tween - polysorbate-type nonionic surfactant, Poloxamer - triblock PEO–PPO–PEO copolymers of poly(ethylene oxide) (PEO) and poly(propylene oxide) (PPO), Soluphor P - (2-Pyrrolidone), Transcutol (2-(2-ethoxyethoxy)ethanol), Transcutol-P (diethylene glycol monoethyl ether), vitamin E-TPGS - D-α-tocopheryl polyethylene glycol succinate

### 2.1 Oil Phase

Ophthalmic nanoemulsions contain from 5 to 20 wt % of oil/lipid as the dispersed phase. The selection of the lipid phase is an important aspect in the design of nanoemulsions, as API is dissolved in an oil prior to the dispersion in an aqueous phase. The selection of the oil phase for nanoemulsion formulation is frequently based on the solubility of API in different oils.^9^ In addition, the oil used in the formulation needs to be well tolerated and compatible with the other excipients included in the nanoemulsion. The following compounds are frequently used to prepare ophthalmic nanoemulsions (Table 2): vegetable oils, glycerides, medium-chain triglycerides, long-chain unsaturated fatty acids, and polyalcoholic esters of medium-chain fatty acids. Vegetable oils administered topically to the eye include: soybean oil, castor oil, peanut oil, olive oil, jojoba oil, and Babchi seed oil.^30,35,36^ Medium-chain triglycerides such as Miglyol 812, Captex 355, 200 or 8000, Witepsol, and Labrafac as well as long-chain unsaturated fatty acids (oleic and octanoic acids) are also used as an inner phase of nanoemulsions.^30,34−36^ From the group of polyalcoholic medium-chain and long-chain fatty acid esters, the following are used: ethyl oleate, isopropyl myristate and isopropyl palmitate. Triacetin and vitamin E are also mentioned among the ingredients of the nanoemulsion applied to the eye, and additionally, these two compounds in ophthalmic formulations can act as humectants and antioxidants.

**Table 2:** Selected Properties of Lipids Used in the Formulation of Ocular Nanoemulsions

oil phase component	surface tension at 20 °C [mN/m]	dynamic viscosity at 20 °C [mPa·s]	density at 20 °C [g/cm^3^]	refractive index [nD] at 20 °C	ref
castor oil	39.0	950–1100	0.955–0.968	1.477–1.479	(41−43)
corn oil	31.6 at 23 °C	31 at 40 °C	0.915–0.918	1.474–1.476	(42−45)
coconut oil	33.4	39 at 30 °C	0.917	1.448–1.450 at 40 °C	(42,43)
soybean oil	25	50.09 at 25 °C	0.916–0.922 at 25 °C	1.470–1.478	(43,45)
evening primrose oil	-	-	0.926 at 25 °C	1.479	TDS^a^
linseed oil	-	-	0.928–0.933	1.479–1.481	(41,45)
liquid paraffin	35 at 25 °C	110–230	0.827–0.89	1.476–1.480	(43)
olive oil	31.9 at 23 °C	80	0.914	1.467–1.471	(41,42,44)
peanut oil	31.3 at 23 °C	68–77	0.912–0.920	1.460–1.472	(41,42,44)
oleic acid	32.79	26 at 25 °C	0.895	1.458 at 26 °C	(43)
isopropyl myristate	29.7	5–7 at 25 °C	0.850 at 25 °C	1.434	(43)
glyceryl triacetate	36.5	17.4 at 25 °C	1.160 at 25 °C	1.429	(43)
Miglyol 812	25–33	-	0.93–0.96	1.449–1.451	TDS^a^
transcutol	31.8 at 25 °C	3.85 at 25 °C	0.999 at 25 °C	1.427	(46)
alpha tocopherol	-	-	0.947–0.951	1.503–1.507	(43)
ethyl oleate	32.3 at 25 °C	3.9 at 25 °C	0.870 at 25 °C	1.451	(43)
Eutanol G	-	58–64	0.835–0.845	1.453–1.455	TDS^a^

a TDS - Technical Data Sheet.

Furthermore, in order to obtain transparent formulations with reduced viscosity of the dispersed phase a mixture of oils can be used (an example includes the combination of castor oil with medium chain triglycerides in 1:1 ratio, resulting in a decrease of castor oil viscosity).^34^ The composition and viscosity of the oil phase in nanoemulsions affect the size of the obtained droplets. It was shown that high-viscosity oils (above 3.5 mPa·s) produced larger droplets as compared with oils of lower viscosity. To obtain nanoemulsions with fine droplets an optimum viscosity ratio between the dispersed (ηd) and the continuous phase (ηc) of 0.05 ≤ ηd/ηc ≤ 5 was proposed. In cases of very thick oils, droplet size can be reduced by increasing viscosity of the continuous phase.^37,38^

### 2.2 Surfactants and Cosurfactants

The important components of nanoemulsions affecting their physical stability are surfactants and cosurfactants, enabling a successful emulsification of the oil into the continuous phase. The surfactant should be soluble in the continuous phase of the nanoemulsion, ensure very low interfacial tension, and prevent coalescence of the oil droplets during homogenization.^39^Table 3 provides HLB values of selected surfactants used in ocular nanoemulsions. To obtain a 20 wt % o/w nanoemulsion, a 5–10 wt % surfactant concentration is required. In preparations for application to the eyeball low-toxicity nonionic emulsifiers are most commonly used.^30^ This group includes: polysorbates (Tween), sorbitan esters (Span), poloxamers (Pluronic F68, Pluronic L - 62TM, Pluronic F127), polyoxyethylene fatty acid esters (Emulphor), hydrogenated castor oil derivatives (Kolliphor EL, Kolliphor RH40, Kolliphor RH60), Tyloxapol, Solutol HS 15, Vitamin E-TPGS, and polyethylene glycol succinate. The analysis of the literature shows that in order to obtain a stable nanoemulsion it is more advantageous to combine nonionic and cationic emulsifiers.^34^ The most commonly used cationic surfactants in ophthalmology are stearylamine and oleylamine; however, both form emulsions with low stability. The positively charged nanoemulsions also contain preservatives such as cetrimide, benzalkonium chloride, benzethonium chloride, cetylpyridinium chloride, cetalkonium chloride, and benzododecinium bromide, which have surface active properties. These preservatives in conventional eye drops may be irritating to the eyeball. However, in emulsions they concentrate in the oil phase, which reduces their release and toxicity.^40^

**Table 3:** HLB Value of Surfactants Frequently Used in Ocular Nanoemulsion

surfactant	HLB value	ref
Brij 35	16.9	(50)
Span 20 (sorbitan monolaurate)	8.6	(50)
Span 40 (sorbitan monopalmitate)	6.7	(50)
Span 80 (sorbitan monooleate)	4.3	(50)
Tween 20 (PEG-20 sorbitan monolaurate)	16.7	(50)
Tween 40 (PEG-20 sorbitan monopalmitate)	15.6	(50)
Tween 80 (PEG-20 sorbitan monooleate)	15.0	(50)
Kolliphor RH60 (Polyoxyl 60 hydrogenated castor oil)	15–17	(43)
Poloxamer 188 (Pluronic F68)	29.0	(51)
Poloxamer 407 (Pluronic F127)	22.0	(51)
Tyloxapol	13.0	(49)
Soluphor P	12–14	(52)

The amphoteric emulsifiers used in the eye drops include: lecithin obtained from soybean or chicken eggs and its fractions as well as amphoteric surfactants (e.g., Miranol MHT and Miranol C2M conc NP).^30,34^ In order to increase the miscibility of both phases and to ensure the interface fluidity, the addition of amphiphilic compounds, the so-called cosurfactants, may be required. The most frequently used cosurfactants in ocular nanoemulsions include short- and medium-chain alcohols (e.g., ethanol, benzyl alcohol, glycerol, propylene glycol, and PEG 400).^39,47^

### 2.3 Water Phase

The composition of the water phase may also affect the droplet size and the stability of the nanoemulsion. When preparing nanoemulsions, the pH value and the presence of electrolytes and ions should be examined, as they may affect the size of the dispersed phase and the stability of the formulation.^47,48^ The most commonly used water phase in ophthalmic preparations is pharmacopoeial water for injections or buffered saline solution.^16,40^

### 2.4 Other Auxiliary Substances

Ophthalmic formulations require addition of other excipients enabling their application to the eyeball, including preservatives, buffers, osmotic pressure, and viscosity modifiers, humectants, or gelling agents. The following preservatives are used in ocular nanoemulsions: benzalkonium chloride (0.01–0.1% w/v), cetrimide (0.01–0.1% w/v), chlorocresol, parabens, and alcohols (e.g., chlorobutanol and phenoxy-2-ethanol).^34,38^ If buffering is necessary, citrate, phosphate, or borate buffers are used.^49^

The osmotic pressure of ophthalmic nanoemulsions is adjusted by the addition of mannitol (0.15–0.3% w/v), glycerol (2.5–5% w/v), sorbitol, propylene glycol, and dextrose.^30,46,50^ The viscosity of the ocular nanoemulsions can be increased by the addition of natural and synthetic polymers which have strong water-absorbing properties and create viscoelastic gels. As a result, a prolonged contact time of the drug with the cornea can be achieved which, in turn, may increase its bioavailability. For this purpose polysaccharide polymers such as methylcellulose, hydroxyethylcellulose, hydroxypropylcellulose, hydroxypropylmethylcellulose, gellan gum, xanthan gum, hyaluronic acid as well as synthetic polymers such as poly(vinyl alcohol), polyvinylpyrrolidone, carbomeric (weakly all amic acid), and polyethylene glyceric acid can be used.^53,54^

## 3 Selection of Nanoemulsion Ingredients—Ternary Phase Diagram

The Gibbs phase rule is used to define the composition of three-component nanoemulsions. The rule specifies number of intensive variables (degrees of freedom, e.g., number of phases in the system, number of independent components) that can be varied independently without disturbing the number of phases in equilibrium.^55^ This, in principle, can be used to determine the formulation space which can be modified without affecting the quality of the final product. For thermodynamic systems containing three liquid components, a ternary phase diagram (the so-called Gibbs phase triangle) enables us to conclude whether, at a given temperature and pressure, selected amounts of these components will form single phase, separate into two immiscible solutions or whether these compounds will not mix with each other.

**Figure 3:**
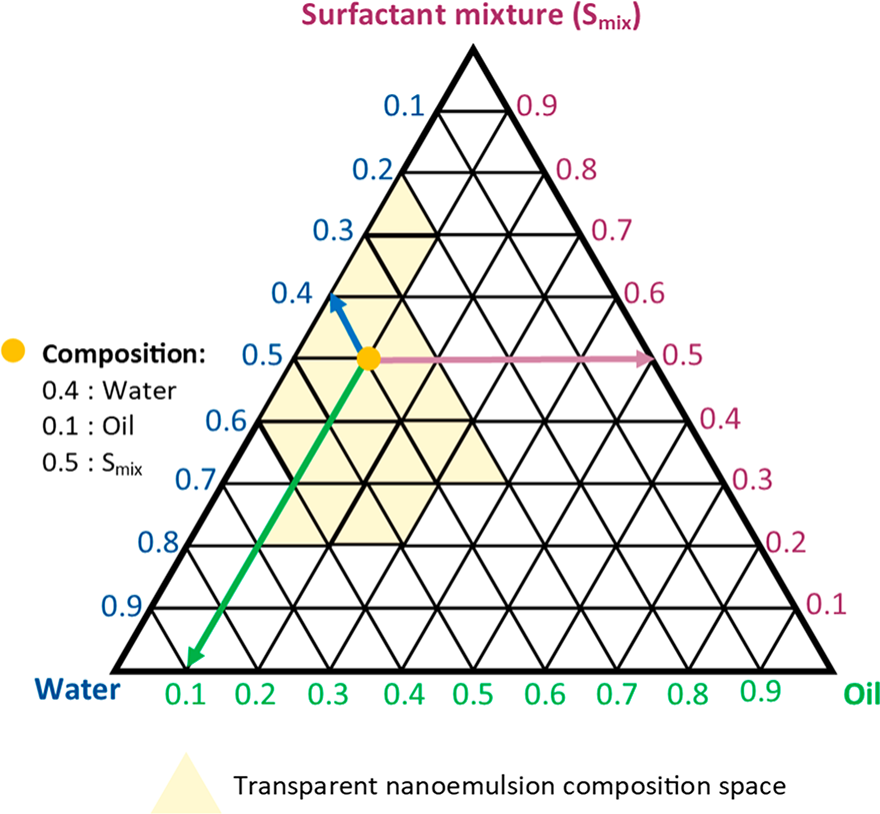
An example of a ternary phase diagram plotted for an oil, water, and a mixture of emulsifiers. The light yellow field on the graph indicates the formation of a transparent nanoemulsion, stable at the 24 h mark. Yellow point indicates an example of a nanoemulsion composition.

In the case of nanoemulsions, a pseudoternary phase diagram is constructed, where component A is an oil, component B is water, and C is a mixture of surfactant and cosurfactant (Figure 3). The point on the side of the triangle opposite the vertex (e.g., Water) represents the percentage composition of the binary system (e.g., Oil+Surfactant mixture, Figure 3).

In turn, the point located in the inner area of the triangle describes the composition of the ternary system. The amount of each component in the system can be defined visually by plotting a line parallel to each side of the triangle. Therefore, the entire inner space of the triangle is divided into a set of small equilateral triangles that describe the formulation space of the emulsion.^56^ The construction of the diagrams enables determination of the formulation space where a clear nanoemulsion is most likely to form and to optimize the proportion of an oil, water, and a mixture of surfactant and cosurfactant in the formulation. Shafiq-un-Nabi et al. constructed a three-component diagram using the water phase titration method.^53,54^ In this method specific volumes of the surfactant and cosurfactant (S_mix_ in different ratios from 1:9 to 9:1 V_surfactant_: V_co-surfactant_) were mixed with different volumes of the oil (in the ratios from 1:9 to 1:0.11 V_oil_: V_Smix_, respectively) following the addition of a predetermined amount of water in the range of 5–95% of the total formulation volume. After the addition of a water aliquot the formulation was thoroughly mixed and left for 24 h to equilibrate.^53^

On the basis of visual observation the formulations were divided into 4 categories: 1. clear and easy-flowing nanoemulsion, 2. clear gel, 3. milky emulsion, 4. milky emulgel.^53^ The compositions of clear and transparent single-phase mixtures obtained after equilibration were marked as points on the phase diagram. The area covered by these points represented the composition region enabling formulation of the nanoemulsion.

## 4 Technological Methods of Producing Ophthalmic Nanoemulsions

On the basis of the amount of energy used in the technological process, nanoemulsion formulation methods can be divided into low-energy and high-energy methods. Low-energy methods include spontaneous emulsification, solvent evaporation, phase inversion temperature (PIT) method or solvent displacement method, whereas high-energy methods include high-energy mixing (high-energy stirring), high-pressure homogenization (HPH), microfluidization, membrane emulsification, ultrasonication, or jet homogenization (see Table 4 for comparison between low- and high-energy methods; see SI Table S1 for nanoemulsions formulations examples obtained by low-energy and high-energy methods and Table S2 for nanoemulsions evaluated in clinical trials).^57^

**Table 4:** Comparison of Ophthalmic Nanoemulsions Preparation Methods^57−59^

feature	low-energy methods	high-energy methods
energy input	- 10^3^–10^5^ W/kg	>10^8^ W/kg for droplets diameter <100 nm
specialized equipment	- generally not required	- high-pressure homogenizers
		- microfluidizers
		- ultrasonicators
		- stream homogenizer
pressure	- not applied	- high-pressure homogenization method: 500–5000 psi
		- microfluidization: 500–20 000 psi up to 50 000 psi
		- stream homogenizer: 43 500–58 000 psi
temperature	- wide range of temperatures can be used in nanoemulsions formulation (except fixed temperature in PIT method)	- local temperature increases during the process, not suitable for thermolabile drugs
	- suitable for thermolabile drugs (except the PIT method)	
	- it allows the protection of sensitive compounds from the harsh conditions of the high-energy methods, especially temperature and pressure	
droplet size and size distribution	- up to 50 nm	- ultrasonic emulsification allows to obtain emulsion droplets with a size of 200 nm
		- in microfluidization narrow size distribution of particles and smaller particles of the dispersed phase are obtained as compared to conventional homogenization methods
		- in the high-pressure homogenization method, the particles diameter of the dispersed phase reaches sizes close to 100 nm
production cost	- low production costs	- high costs associated with the purchase of the equipment and higher energy consumption in the production process

### 4.1 Spontaneous Emulsification or Self-Emulsifying Methods

**Figure 4:**
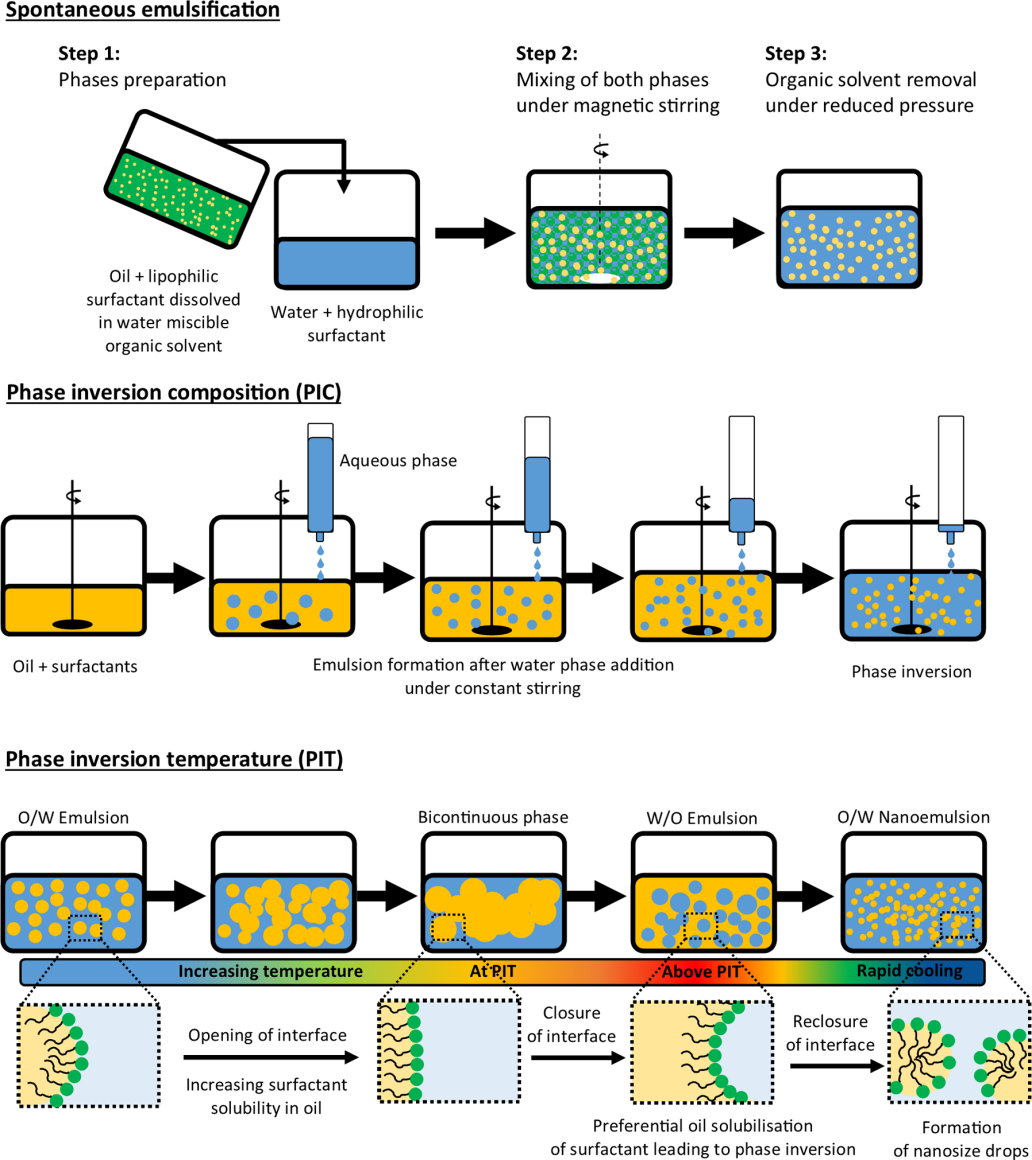
Schematic representation of low-energy methods used in the formulation of ocular nanoemulsions. From the top: spontaneous emulsification, phase inversion composition, and phase inversion temperature.

The spontaneous emulsification has been the most frequently described method for the preparation of ophthalmic nanoemulsions. In this method the water phase is added in portions to the mixtures of an oil with the preselected surfactants and cosurfactants at room temperature under gentle stirring (Figure 4). Preparation of nanoemulsions using the spontaneous emulsification method takes place in 3 stages: in the first stage two phases are prepared, a homogeneous lipid phase consisting of an oil and a lipophilic surfactant in a water-miscible solvent and a second phase formed by water and hydrophilic surfactant. In the second step an o/w emulsion is instantaneously formed during the addition of the lipid phase to the water phase with continuous magnetic stirring, and finally, in the third step, the water–miscible solvent is removed by evaporation under reduced pressure.

As a result, nanodroplets of the oil are dispersed in an aqueous solution of water and hydrophilic surfactant.^14,35,57,58^ The process of nanoemulsions’ formulation using the self-emulsification method utilizes the chemical energy released during diluting the inner phase with a continuous phase, usually at a constant temperature without any phase changes during emulsification. The formation of nanoemulsions using spontaneous emulsification method is a two-step process. First, a bicontinuous microemulsion is formed at the interface between the organic and the aqueous phase, following its disorganization, leading to spontaneous generation of fine oil droplets. The bicontinuous microemulsion can only be formed using a certain range of surfactant–oil–water ratios depending on the components of the nanoemulsion. Application of mild stirring may facilitate the breakdown of the bicontinuous phase because of increased mixing of the surfactant, oil, and water molecules.^58^ The formulations prepared as described, although thermodynamically unstable, may have high kinetic energy and long-term colloidal stability.^28,39^ This method enables formulation of the particles of the dispersed phase with the diameter of ca. 50 nm. The size of the nanoemulsion drops formed in this process is influenced by several factors, including the amount and type of both surfactant and cosurfactant, the ratio between the surfactant and the dispersed phase, the presence of additives in the dispersed phase as well as the composition, and the viscosity of both phases.^59^ The spontaneous emulsification method for nanoemulsions’ formulation has several advantages as compared with high-energy methods and low-energy methods (e.g., the PIT method). It allows for protection of the labile compounds against the harsh conditions of the high-energy method, especially the temperature and pressure. Furthermore, in comparison with other low-energy techniques, it allows reduction of the surfactant content while ensuring thermal stability.

### 4.2 Emulsification and Solvent Evaporation Method

Ophthalmic nanoemulsions can also be obtained by spontaneous emulsification method following evaporation of a water-miscible organic solvent in which the oil phase is dissolved. In this method a mixture of solvent and oil at room temperature is dispersed in the aqueous phase in which a surfactant is present. After mixing, the organic solvent rapidly diffuses into the water which causes the oil to disperse in the form of nanosize droplets. At the end of the process, the solvent is evaporated from the emulsion under reduced pressure.^60,61^

In their patent application Valdivia et al. described an o/w nanoemulsions formulation method for ophthalmic use. In this method, an API is dissolved in an oil phase, which is further dissolved in a water-miscible organic solvent with a dielectric constant greater than 15 to form an organic phase. In the next step, the organic phase is added to the aqueous phase with moderate agitation to form a nanoemulsion, followed by removal of the organic phase and part of the water phase under reduced pressure at a temperature below 35 °C. Using this method a homogeneous nanoemulsion with droplets with an average particle diameter of about 200 nm can be obtained.^62^

### 4.3 Phase Inversion Method

The low-energy methods also include the phase inversion method where the inner phase is dispersed within the continuous phase due to the changes in the formulation composition (phase inversion composition method, PIC) or temperature (PIT) (Figure 4). The phase inversion point can be defined as the point at which the surface tension between the water and oil phases of the emulsion (ca. 10 μN/m) enables the spontaneous formation of nanosize droplets without any energy input.^63^ In the phase inversion methods one can distinguish either transient or irreversible inversion of the system. The transient phase inversion can be caused by temperature or electrolyte concentration changes which, in turn, affects the HLB of the formulation. Simultaneously irreversible phase inversion takes place at a constant temperature and is caused by a change in the composition of the emulsion.^48,59^ Transient inversion takes advantage of the difference in solubility of emulsifiers in water or in oil at different temperatures which induces a conversion of a w/o emulsion to an o/w emulsion or vice versa. In the first step of this process a macroemulsion is heated to a temperature corresponding to the phase inversion point of the formulation following rapid cooling of the mixture to 25 °C during the second step. The increase in temperature leads to the opening of the surface film at the interface between two phases and their inversion, followed by a rapid decrease in temperature which closes the interfacial structure of droplets. The dispersed nanoemulsion droplets remain stable over an extended period of time due to their surfactant coating. In general, good mutual solubility of water, oil, drug substance, and surfactant facilitates the phase transition in this method. The main limitation of this method is its restricted applicability to thermolabile substances.^38,63^

### 4.4 High-Energy Methods

High-energy methods enable controlling the size of the dispersed phase particles via different technological processes, homogenization conditions (e.g., time, temperature), as well as the properties and composition of the starting mixture. For the formulation of nanoemulsions several different techniques including high-pressure homogenization, microfluidization, or ultrasonication have been applied.

**Figure 5:**
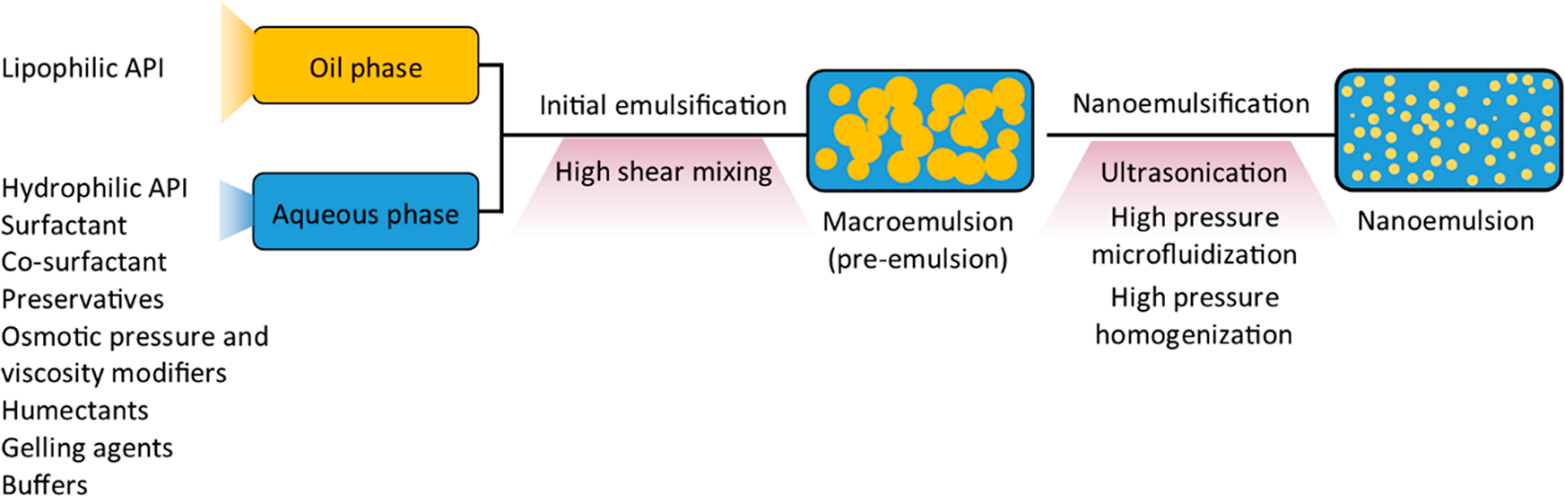
General formulation approach of nanoemulsions using high-energy methods.

**Figure 6:**
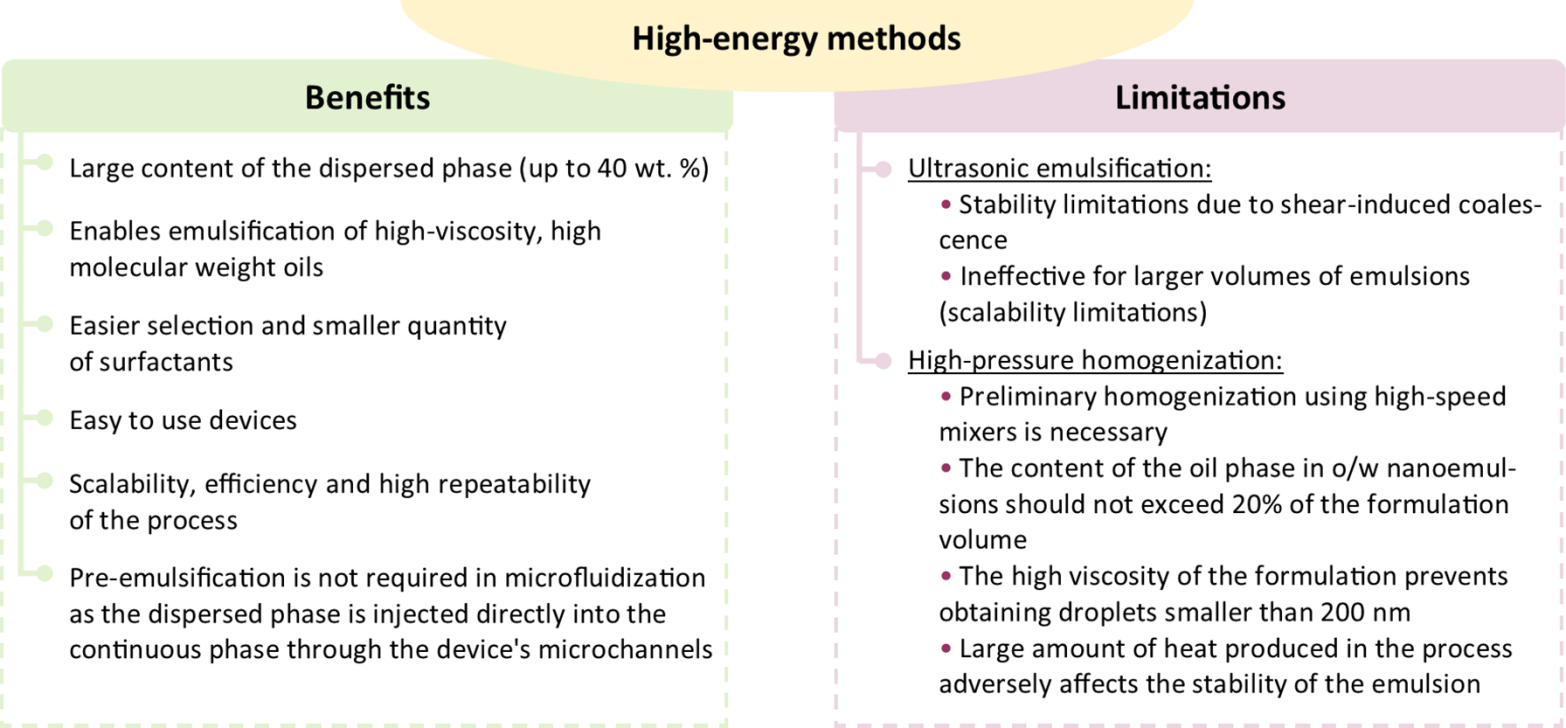
Benefits and limitations of high-energy techniques for nanoemulsions formulation.

The high energy input required in these methods may induce a local increase of the temperature within the formulation which makes them not suitable for the homogenization of thermolabile compounds such as retinoids and macromolecules (proteins, enzymes, or nucleic acids). Furthermore, the high energy consumption and required access to the specialized equipment increase the production cost of the nanoemulsions (see Figures 5 and 6).^59^

#### 4.4.1 High-Pressure Homogenization

**Figure 7:**
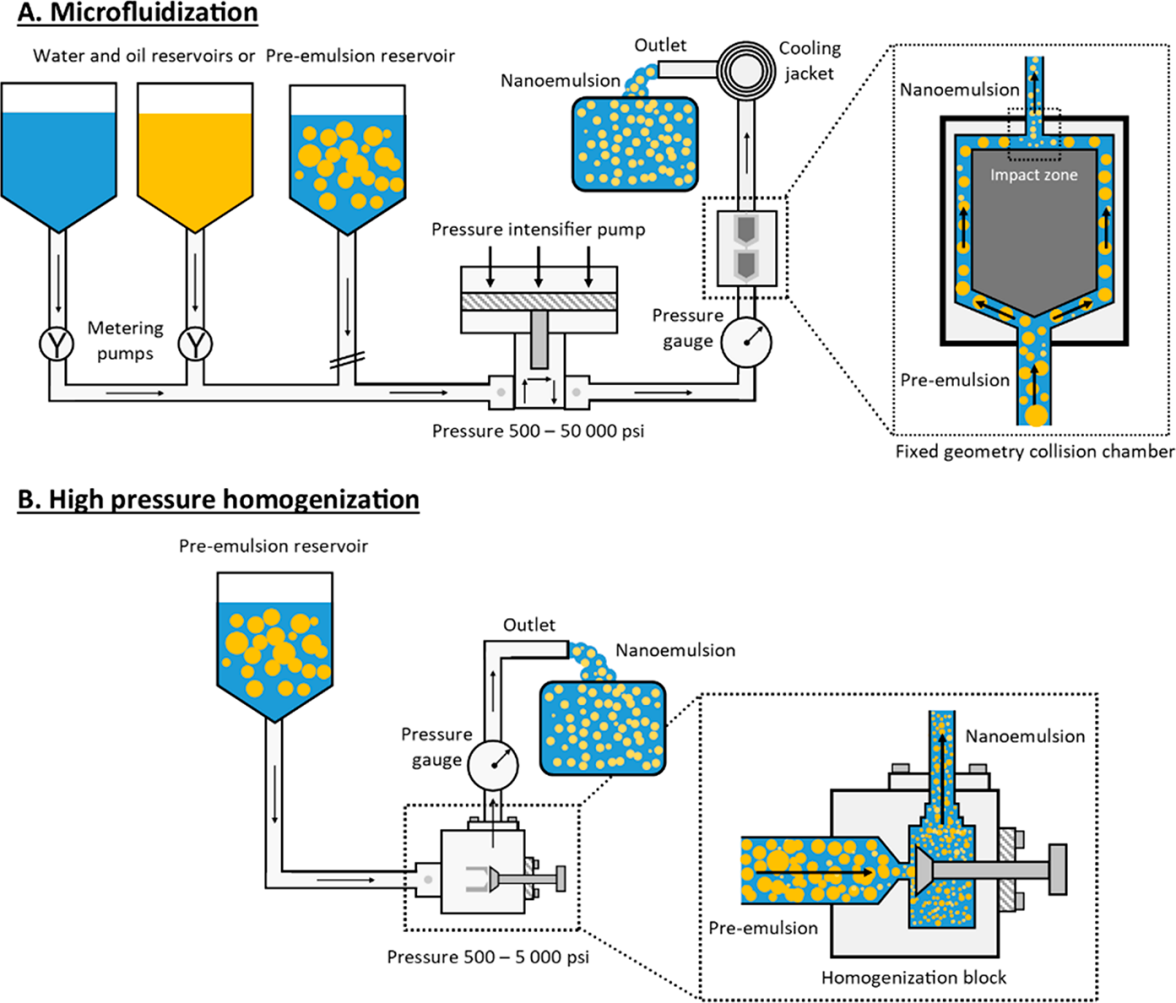
General formulation approach of nanoemulsions using high-energy methods: A. Microfluidization, B. High-pressure homogenization.

The general protocol for the preparation of the o/w nanoemulsions using high-energy methods begins with initial homogenization of a mixture of an oil, a surfactant, and water with a high-shear mixer to form a macroemulsion (pre-emulsion). In the second step the resulting macroemulsion is homogenized with a high-pressure homogenizer using hydraulic shear, intense turbulence, and cavitation (see Figures 5 and 7B).

In a high-pressure homogenizer the two immiscible liquids with the addition of emulsifiers pass through a piston gap with a height on the order of a few microns^58^ under high pressure (500–5000 psi or 35–445 bar), resulting in a homogeneous nanoemulsion with a particles’ diameter of dispersed phase of about 100 nm.^34,47,58,64^ The mixture is typically processed multiple times through the homogenizer, and the final droplet size depends on the number of homogenization cycles and the composition of the formulation. The most important advantages of high-pressure homogenization in the industrial production of nanoemulsions are high efficiency, scalability, and repeatability of the process.

The major limitations of this method are the unfavorable coalescence process, which can be reduced by addition of surfactants in excess,^47^ and relatively low content of the oil phase enabling the formulation of the o/w nanoemulsions which should not exceed 20 vol % of the formulation.^59^ High-pressure homogenization (HPH) can be carried through two techniques, the hot and cold HPH. While both techniques have their advantages, cold high-pressure homogenization is used for extremely temperature-sensitive compounds. In the hot HPH technique, an API is dissolved or dispersed in the lipid phase which is further dispersed in the surfactant solution heated above its melting point by vigorous stirring. The obtained pre-emulsion is then processed using high-pressure homogenization at increased temperature in order to obtain a highly homogeneous nanoemulsion. In contrast, in the cold HPH technique, the mixture of API and the lipid phase is cooled below the solidification point of the lipid, ground, and dispersed in a cold surfactant solution resulting in formation of a micronized presuspension. The obtained presuspension is processed through a high-pressure homogenizer at room temperature to obtain a homogeneous nanoemulsion. Both techniques require homogenization cycles optimization in order to obtain narrow droplet size distribution. It has been shown that low polydispersity of a formulation can be secured by increasing the number of homogenization cycles. On the contrary, an increased number of cycles can reduce the efficiency of the process and affect the quality of the product due to the generation of large heat amounts which is indicated as a limitation of the high-pressure homogenization technology. Using this method Kotta et al. successfully procured o/w nanoemulsions containing less than 20 wt % of the oil phase.^65^ An additional limitation of nanoemulsions preparation using this method may be the high viscosity of the system which prevents obtaining the inner phase droplets with sizes smaller than 200 nm. Gallarate et al. used high-pressure homogenization to obtain an o/w ophthalmic nanoemulsion with timolol. The authors prepared a pre-emulsion by mixing the formulation components for 10 min at 15 000 rpm in a mechanical homogenizer followed by 3 cycles of high-pressure homogenization, 5 min at 1000 bar each.^66^ Benita and co-workers at Novagali Pharma S.A., France (now Santen SA, Geneva, Switzerland) developed a cationic nanoemulsion (Novasorb) using a three step process. The first step involved mixing the oil and water phases with a magnetic stirrer at 100 rpm and with a high-shear stirrer at 16 000 rpm afterward to obtain an emulsion with oil droplet size of about 1 μm. The emulsion was subjected to high-pressure homogenization (pressure of 1000 bar and temperature 4 °C) resulting in droplet sizes of 150–200 nm.^9,67,68^

#### 4.4.2 Microfluidization

The microfluidization method is also used for preparation of nanoemulsions and is based on two immiscible phases of the system being fed from two opposite microchannels, colliding with each other in the impact zone of a high-pressure positive displacement pump under pressure of 500–20 000 psi (from about 35 to about 1380 bar) up to 50 000 psi (Figure 7A).^47,58,59^ Droplet size reduction in the microfluidizer is achieved via combined use of a hydraulic shear, impact, attrition, impingement, intense turbulence, and cavitation. Droplet size of the dispersed phase obtained in this method depends on the applied pressure, the number of passages in the chamber of the device, and the formulation composition. The microfluidization is also a direct emulsification process as it does not require pre-emulsification. The dispersed phase is injected directly into the continuous phase through the microchannels instead which presents the advantage over the high-pressure homogenization method. From a mechanical point of view, a microfluidizer is a static mixer with no moving parts, enabling production of small droplets of the dispersed phase with narrow size distribution. Furthermore, microfluidization can be used for both laboratory- and industrial-scale formulations. Dukovski et al. successfully used a microfluidizer to prepare uncoated and chitosan coated ibuprofen nanoemulsions. For uncoated nanoemulsions, the oil phase composed of ibuprofen dissolved in a lecithin solution in Miglyol 812 was first premixed with water solution of Kolliphor EL using a magnetic stirrer followed by its homogenization with a high-shear stirrer at 6000 rpm for 5 min. The obtained pre-emulsion was processed in a microfluidizer for 5 cycles under the pressure of 1000 bar. The chitosan-coated nanoemulsions were in turn produced by the addition of the chitosan solution to the aqueous phase before mixing and processing in a microfluidizer (1000 bar, 5 cycles).^69^

#### 4.4.3 Ultrasonication

**Figure 8:**
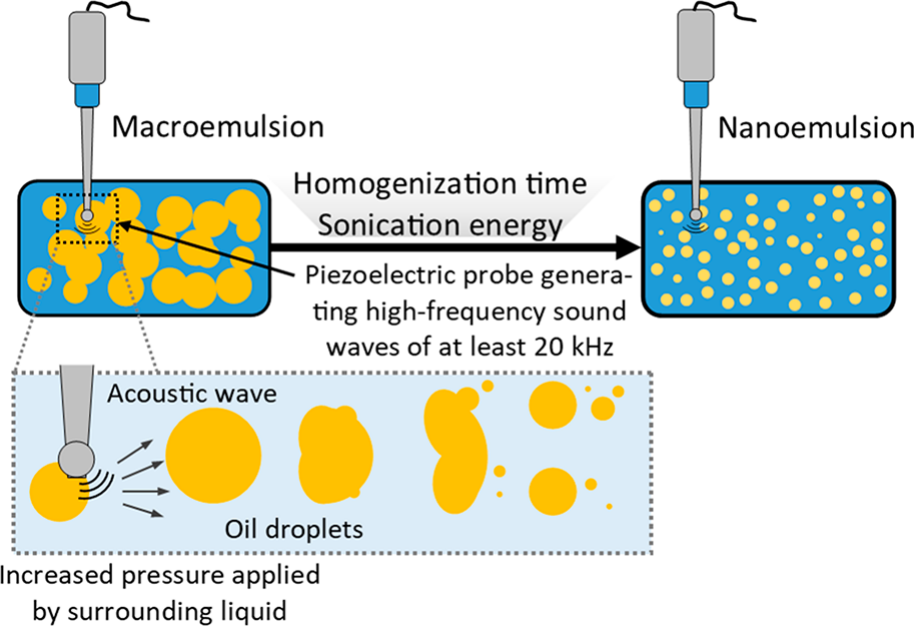
Reduction of droplet size of an emulsion via ultrasonication.

During ultrasonic emulsification the energy necessary to break the droplets of the inner phase of the emulsion is provided by a sonicator probe (sonotrode) that emits high-frequency sound waves of at least 20 kHz (Figure 8). Sonotrodes contain a piezoelectric quartz crystal that can expand and contract in the solution depending on the applied alternating voltage. As the tip of the ultrasonic probe comes into contact with the liquid, it produces mechanical vibration causing cavitation (i.e., the formation and collapse of voids in the liquid induced by local reduction of pressure to or below the vapor pressure of the liquid). The expansion forces generated by the sound wave during the expansion phase trigger the disruption of the liquid structure. Cavitation results in the formation of microjets (acoustic jets), shear stresses, shock waves, and turbulence in the fluid medium. Emulsification with the use of ultrasound is a two-stage process. Initially, waves breaking up the dispersed phase particles are formed in the acoustic field, and then acoustic cavitation occurs.

When the acoustic wave propagates in the liquid causing its temporary local thinning and pressure drop, it favors the formation of microbubbles and their subsequent compaction and dispersion into smaller droplets due to pressure fluctuations. The droplet formation of the nanoemulsion is controlled by the interaction between droplet breakdown and droplet coalescence. These devices are mainly used in laboratories to obtain emulsion droplets with a size of 0.2 μm.

The probe in the ultrasonificator exposed to the formulation produces mechanical vibrations and cavitation, which provides the energy necessary to create small-sized droplets.

Obtaining fine droplets of the dispersed phase in an ultrasonication process requires optimization of emulsifier type and concentration as well as viscosity of continuous and dispersed phases.^70^ The particle size of the dispersed phase in nanoemulsions achieved by the use of ultrasound decreases with increasing homogenization time and sonication power along with the increase of the surfactant concentration in the formulation. Shear-induced coalescence may be an unwanted effect of this method.^58^ Goel et al. used a sonicator to prepare a nanoemulsion with natamycin. Formulation components were premixed first at 2000 then at 16 000 rpm for 5 min with a stirrer following ultrasonication at a 40% amplitude for 10 min with a 10-s pulse turned on and a 15-s pulse turned off.^71^ Sonication is a popular method used to produce nanoemulsions on a laboratory scale; however, it is not so widely used on an industrial scale, as it is ineffective for larger volumes of emulsions.^41^

#### 4.4.4 Jet Homogenization Method

High-energy methods also include homogenization using a jet homogenizer in which two or more streams of emulsions delivered from opposing nozzles with a hole diameter of 0.3–0.5 mm collide with each other under high pressure. The jet homogenizer uses high pressure from 300 to 400 MPa at which the laminar air flow breaks the droplets to a small size. In the outlet valve the emulsion droplets are broken down in three stages; initially the droplets in front of the valve are lengthened, and then they undergo deformation inside the valve and break via turbulent flow after exiting the valve.^72^

### 4.5 Industrial-Scale Production of Ophthalmic Nanoemulsions

A well-designed, small-scale ophthalmic nanoemulsion manufacturing process may prove to be unreliable or nonreproducible on a larger scale if not effectively controlled during the up-scale of production. The scaling-up of the emulsification process and the examples of large-scale nanoemulsion production are still limited. In most cases the loss of product stability after upscaling is due to the inability of industrial manufacturers to apply the same shear forces that were used in the preformulation phase. Therefore, the selection of the formulation technology and robust equipment for large-scale production is fundamental. Factors that may change during scaling-up include, but are not limited to, structural changes of API and auxiliary substances, changes in particle size of the dispersed phase, variations in drug content along the quality, and quantity of impurities. Furthermore, the changes in manufacturing processes can lead to reduced stability or even degradation of the product. On the laboratory scale, once the nanoemulsion composition and shear forces are optimized, the average size of the droplets of the dispersed phase can be reduced to the nanometer range. The same level of stability during industrial production can only be achieved if the average droplets’ size of the oil phase remain unchanged.

While obtaining a laboratory-optimized defined composition of nanoemulsion formulation on an industrial scale is usually uncomplicated, maintaining the required intensity of shear forces is much more challenging. High-pressure homogenization, microfluidization, and high-amplitude ultrasonic treatment are currently the leading methods used to produce the highest-quality nanoemulsions. Despite their disadvantages, for example, the need to prepare a droplet dispersion (pre-emulsion) with a size of 1–10 μm using a rotor-stator colloid mill, HPH and microfluidization are currently the preferred technologies for industrial production of pharmaceutical nanoemulsions. Both processes are energy-intensive, require high-maintenance (both cleaning and wear) expensive equipment, and a significant process redesign to enable aseptic production.^63^ Microfluidization provides some advantage over HPH because of the construction of the equipment that enables easier scale up by multiplication of microfluidization chambers. This, in turn, provides nanoemulsions with similar properties (globule size, PDI) as compared to formulations obtained at a laboratory scale. Furthermore, the equipment manufacturing companies provide solutions that enable scaling the nanoemulsion formulation process from laboratory through pilot to production scale. The available industrial microfluidization equipment also enables sterile production and packing in a single process.

The ultrasonic nanoemulsification is also possible to scale-up. In the process where the driving force is acoustic cavitation generated by an ultrasonic homogenizer, the higher the ultrasound amplitude, the higher the production speed, and the better quality of the final product. However, scaling-up the process requires enlarging the cavitation zone without losing its intensity. Industrial ultrasonic processors contain much larger ultrasonic sonotrodes than laboratory devices (larger ultrasonic horns) and are capable of generating larger cavitation zones; therefore, they process much more material per unit of time as compared with laboratory sonicators. Furthermore, scaling-up the process requires the same cavitation intensity in the production environment as originally used in the laboratory. It means that to obtain reproducible results after scale-up, the sonotrode amplitudes in laboratory and industrial processors must be kept at the same level. Conventional industrial ultrasonic devices cannot provide high enough amplitudes for efficient nanoemulsification that compromise the product quality. Newly developed industrial ultrasonic devices that utylise so-called Barbell Horn Ultrasonic Technology (BHUT) provide the same high ultrasonic amplitudes and cavitation intensity as used during the laboratory phase of product development, enabling the achievement of reproducible results on an industrial scale.^63,73^

### 4.6 Strategies for Sterile Production of Nanoemulsions

The final product of a nanoemulsion can be sterilized by the means of double filtration,^74^ moist heat terminal sterilization (121 °C for 15 min under 15 psi pressure),^69,75^ or through aseptic production of the emulsion.^13,76^ As the sterilization process has a great impact on the formulation physical integrity, it requires careful consideration as far back as the early stages of the formulation development.^77^ The influence of a particular sterilization method on the nanoemulsion properties must be assessed each time through evaluation of particle size of the dispersed phase, the polydispersity index, and the zeta potential. Filtration is a commonly used nanoemulsion sterilization method, especially for formulations containing thermolabile substances. However, possible loss of solute as a result of its adsorption on the filter, as well as release of pollutants from the filter must be appraised upon selecting this method.^75^ The U.S. Food and Drug Administration guidelines on sterile drug products produced by aseptic processing published in 2004 define a sterilizing-grade filter as a filter that, when appropriately validated, will remove all microorganisms from a fluid stream, producing a sterile effluent.^13,78^ Validation of the sterile filtration stage can be demanding considering the filter’s pores’ diameter of 0.22 μm, where any particles larger than 220 nm may cause significant filter capacity reduction and, eventually, clog the filter completely.^79^ Thus, the manufactured nanoemulsions require not only a small particle size (the target being smaller than the maximum pore size making the filtration an adequate sterilization method for nanoemulsions), but also a narrow particle size distribution. Furthermore, various pollution sources introduced along production processes can pose an additional challenge to the filtration sterilization process. Additionally, both filtration and aseptic preparation of the formulation require an aseptic method of loading the final formulation container which makes the aseptic processes very expensive. Autoclaving the final formulation in the target container when all possible alterations are excluded has a significant advantage over other sterilization methods as it eliminates the risk of external contamination, also of production origin. The moist heat thermal sterilization is used solely for highly stable emulsions and therefore requires a careful excipient selection as mentioned above.

## 5 Ocular Nanoemulsions Research Methods

Several characterization methods have been developed to assess the properties and the quality of ophthalmic nanoemulsions. These methods enable investigating the physicochemical properties of the formulation (e.g., visual appearance and transmittance, particle size distribution and polydispersity index, zeta potential, refractive index, pH, osmotic pressure, surface tension, viscosity), its pharmaceutical performance (e.g., determination of the API content and *in vitro* release from a nanoemulsion, assessment of sterility and stability upon storage, investigation of API/formulation ingredients interactions), and biological effects (e.g., mucoadhesive strength, cytotoxicity, irritation, transcorneal permeation, antifungal activity, histological examination of the eye after drug form administration and biodistribution of the drug within eye tissues, pharmacokinetics after administration). As nanoemulsions are complex multicomponent systems, it is necessary to combine complementary characterization techniques in order to understand both the performance and the clinical outcome of the formulation. In this section, the most frequently used nanoemulsions characterization methods are described starting from the determination of physicochemical properties of the formulations through the assessment of their pharmaceutical performance and biological effects *in vivo* and *ex vivo* (*in vitro*, *ex vivo*, and *in vivo* studies of the ophthalmic nanoemulsions formulations are summarized in SI Table S3).

### 5.1 Physicochemical Evaluation of Nanoemulsions

#### 5.1.1 Visual Examination and Transmittance Testing

The visual assessment of nanoemulsions is carried out under diffused daylight over a white and black background, determining the clarity of the tested formulations.^16^ Depending on the size the dispersed phase droplets in relation to the wavelength of light (380 nm < λ < 780 nm), the formulation can be clear with a droplet diameter <50 nm or cloudy with a droplet size range of 50 nm < *d* < 200 nm.^63^ The degree of light scattering in nanoemulsions is a consequence of the amount of the dispersed phase, droplet size, and the refractive index of the dispersed particles. Two types of measuring devices are used to analyze the optical properties of nanoemulsions: UV–vis spectrophotometers and colorimeters.^31^ The UV–vis spectrophotometer measures the transmission or reflection of light in the visible wavelength range from 380 to 780 nm (single-point measurements are performed at 520 nm,^63^ on the basis of which the transmittance of the prepared formulation is determined in percent T (%)). High transmittance of the analyzed formulation (close to 100%) indicates that the developed system is clear and transparent, desirable for the application to the eyeball. The formulations of reduced transmittance have limited application in ocular drug delivery as they may interfere with vision once administered to the eye. During clarity analysis the tested formulation is diluted with a selected solvent and analyzed at the appropriate wavelength (λ_max_) against the reference solvent.^39,80^

#### 5.1.2 Particle Size Distribution of Nanoemulsions

The size of the dispersed particles in the range of 1–500 nm significantly increases the stability of the emulsion after preparation and the contact area of the API with the surface of the eyeball. This in turn may improve the absorption of the drug after application. In addition, the decrease of the dispersed phase droplet size in nanoemulsions increases the penetration of the drug substance into the deeper layers of the eye, including the aqueous humor.^36^ The droplet size and polydispersity of the nanoemulsions are determined at room temperature using the dynamic light scattering (DLS), photon correlation spectrometer (PCS), or microscopic methods.^29^

#### 5.1.3 Dynamic Light Scattering (DLS), Syn. Photon Correlation Spectroscopy (PCS), or Quasi-Elastic Light Scattering (QELS)

DLS is the most frequently used technique for determining the mean particle size of the dispersed phase and the polydispersity index of nanoemulsion systems. In the DLS method the laser beam is scattered on the particles present in the solution, and on the basis of the analysis of changes in light intensity, the average size and the distribution of the particles are determined.^81^ The method used in the measurements monitors the variability of laser light scattering due to Brownian motion of particles as a function of time, with small particles moving through the solution at a higher speed. Measurements of the particle size of nanoemulsions are carried out on concentrated (undiluted) samples or samples diluted with the external phase (i.e., water). Dilution is often necessary when analyzing formulations containing excipients that increase the viscosity of the system, as it hinders the assessment of the droplet size of the dispersed phase.^29,80,82^ Based on the analyzed examples, dilution with deionized water in the range from 40^36^ to 100^82,83^ and even 500^69^ times has been reported. These data do not provide any particular viscosity values that need to be achieved to obtain true value of droplets diameter. Furthermore, particle size measurements of undiluted and diluted formulations may help to distinguish between nano- and microemulsion as dilution will have no effect on the size of nanoemulsion droplets.^84^ While dilution with simulated tear fluid may mimic the nanoemulsions application site one needs to take into account that changes in ionic strength of the formulation may result in aggregation of colloidal droplets and, as a consequence, drastic changes in the obtained particle sizes. The value of the polydispersity index (PDI) determined in the DLS method describes the homogeneity of the particle size distribution of the dispersed phase.^85^ The PDI can range from 0 to 1, with 0 being a monodisperse system and 1 being a polydisperse system. Kumar et al. proposed that PDI values above 0.5 in the case of nanoemulsions indicate their polydispersity.^86^

#### 5.1.4 Microscopic Methods

In order to determine the size, particle morphology, and microstructure of nanoemulsion systems, optical or polarizing microscopes with a high-resolution adapter can be used. Lim et al. determined the morphology of the nanoemulsion dispersion applied to the eyeball using hyperspectral photography from a light microscope with a high-resolution adapter which enables quantitative determination of the size distribution and clustering of nanoparticles in the analyzed formulations.^36^ Hyperspectral imaging is a technique derived from standard digital photography where the image is recorded in three wavelength ranges corresponding to the blue, green, and red channels. A hyperspectral image may consist of hundreds of individual images captured at strictly defined wavelengths, thus providing much more information about objects than traditional digital photography. The possible high spectral resolution of this technique (2 nm and more) allows to present the recorded data as a two-dimensional map of reflection spectra which opens the possibility of quantitative and qualitative analysis of particle size distribution in nanoemulsions. Morsi et al. used a polarizing microscope in order to determine the presence of a liquid-crystal lamellar phase or possible crystallization of the API to assess the stability of nanoemulsions.^30^ Although optical microscopy may provide some insight into structural characteristics of nanoemulsions, a more detailed characterization can be achieved with high-resolution electron microscopy techniques including transmission electron microscopy (TEM), cryoTEM, scanning electron microscopy (SEM), or atomic force microscopy (AFM), enabling the imaging of the dispersed particles with nanometric resolution.^69,83,87^ Dukovski et al. used atomic force microscopy in conjunction with fluorescence microscopy to determine the morphology of the dispersed phase in an ibuprofen-loaded cationic ophthalmic nanoemulsion.^69^ Anjana et al. used TEM to analyze the size and morphology of a curcumin-based ophthalmic nanoemulsion. The imaging was performed directly after drying a drop of 1:100 water diluted nanoemulsion deposited on the film grid.^87^ Shah et al. performed TEM imaging of moxifloxacin nanoemulsions by staining its drops with phosphotungstic acid solution (2% w/v) followed by immobilization on copper grids and drying at room temperature (25 ± 2 °C) prior to analysis.^31^ Although classical electron microscopy techniques (TEM or SEM) require staining and/or drying of nanoemulsions that may affect the size and shape of the nanodroplets, a cryoEM provides the unique oportunity to directly image the obtained colloidal systems without major structural changes. While this method is not yet widely used in ophthalmic nanoemulsion characterization, it will definitely be adopted in the near future, providing novel insight into structure of colloidal nanosize drug delivery systems.^88−90^

#### 5.1.5 Zeta Potential

The zeta potential (ζ) is defined as the difference in electric potential (ΔV) between the dispersion medium (in this case water) and the stationary fluid layer attached to the dispersed oil nanodroplets.^91^ The value of this parameter influences the colloidal stability of the designed nanoemulsions. The measurements of the zeta potential are performed using either undiluted or diluted formulations (e.g., with KCl solution or water with a specific conductivity).^66,92^ Diluting the nanoemulsion with water mimics the effect of the tear fluid after application to the eyeball, although it should be emphasized that the positive charge on the droplet should not change after the dilution.^92^ The zeta potential of neutral nanoparticles in dispersed systems ranges from −10 to +10 mV. The zeta potential greater than +30 mV indicates the presence of strongly cationic nanoparticles while values below −30 mV characterize strongly anionic nanoparticles.^93^ The nanoemulsions’ formulations displaying the zeta potential values above +30 mV or below −30 mV are considered stable. It should be emphasized that the charge of the drops in the nanoemulsion may also affect their absorption after application to the eyeball. It is due to the negatively charged surface of the cornea which binds positively charged particles. This may extend the contact time of the drug with the cornea and increase its bioavailability.^94^

#### 5.1.6 pH Measurement

The pH measurement is performed at 25 °C using the potentiometric method^16^ with electrode calibration using standard buffers with pH 4.0, 7.0, and 10.0.^29,30,86,92,95,96^ The pH of the nanoemulsion should correspond to the physiological value of the tear fluid pH which ranges from 7.0 to 7.4, as it ensures comfortable application of the formulation. Fluids significantly deviating from the acceptable values may irritate the eye, cause excessive secretion of tear fluid, and, as a consequence, rapid rinsing of the API from the conjunctival sac. However, taking into account the buffering capacity of the lacrimal fluid, it is possible to administer solutions with pH values in the range of 3.5 to 8.5 (Ph. Eur. 10.0, 1163). Ammar et al. showed no irritation, good tolerance, and an intact structure of the rabbit cornea after administration of a dorzolamide hydrochloride nanoemulsion with the pH value in the range of 4.34–5.42.^29^

#### 5.1.7 Refractive Index

The refractive index (RI) is an optical property that can be used to describe the isotropic nature of nanoemulsion formulations and to identify chemical interactions between medicinal substances and excipients.^65^ Inhomogeneous distribution of surfactants at the oil/water interface may result in their local ordering and formation of a separate phase (i.e., liquid crystals). Liquid crystals form locally ordered structures with much greater viscosity than bulk nanoemulsions, resulting in the anisotropic optical properties of the formulations. This can be observed as shining when the sample is placed in front of a light source between two crossed polarizers. In contrast, the isotropic nanoemulsions are dark under these conditions.^29,30,85,87,95,96^ In the case of ophthalmic formulations, the determined RI indicates whether the developed formulation affects the quality of vision and the discomfort arising after administration. The refractive index of the tear fluid is in the range of 1.340–1.360, and it is possible to administer eye drops with a maximum RF index of 1.476.^76^

#### 5.1.8 Osmotic Pressure Determination

Osmotic pressure is a colligative property and depends on the number of molecules dissolved in the solution. The physiological osmolarity of the tear film during the day ranges from 231 to 446 mOs/kg.^30^ Formulations which display osmotic pressure below 100 mOsm/kg or above 640 mOsm/kg may cause eye irritation.^97^ However, Haße and Keipert obtained formulations with osmotic pressure in the range of 1200–2400 mOsm/kg, which showed no irritation in the rabbit Draize test.^98^ The measurement of the osmotic pressure is based on the determination of freezing point decrease of an analyzed solution compared to a pure solvent and is performed using an osmometer.^29,30,66,92^

#### 5.1.9 Measurement of Surface Tension

Surface tension is measured using a tensiometer. The method involves measurement of the force needed to detach a submerged Wilhelmy plate or Du Nouy’s ring from the surface of a nanoemulsion at a constant temperature.^29,30^ The physiological value of tear fluid surface tension is in the range of 40–50 mN/m. Nanoemulsions display low surface tension due to the presence of surfactants which enables even dispersion of the oil phase in the water-based media. Furthermore, low surface tension of the formulation may increase the wettability of the cornea and, as a result, the absorption of the drug.^66^ Formulations with significantly lower surface tension, as compared with the tear fluid (i.e., < 35 mN/m) may cause eye irritation, pain, and patient discomfort after application.^29^ In contrast, formulations with high surface tension decrease the stability of the tear film.^99^

#### 5.1.10 Viscosity Measurement

Viscosity of nanoemulsions can be determined at various preset shear rates at 25 °C using a viscometer or rheometers (conical, plate, or capillary).^29,30,47,48,66,85,96,100^ Increasing the viscosity of the eye drops via addition of appropriate auxiliary substances is a frequently used approach to extend the contact time of the drug with the eyeball, thus improving the bioavailability of the drug substance.^92^ The determined viscosity of the physiological tear fluid is approximately 1.5 mPa·s^101^ and the desired viscosity for nanoemulsions applied to the eyeball should not exceed the maximum value of 20 mPa·s because high-viscosity emulsions may block the tear ducts.^29^

#### 5.1.11 Determination of the Active Substance Content

Determination of drug content within a nanoemulsion is carried out via extraction with organic phase. The specified amount of nanoemulsion (e.g., 1 mL) is mixed with an organic phase (e.g., methanol), sonicated, and centrifuged at high speed. The obtained supernatant is analyzed for drug content. To examine the incorporation efficiency of a drug into a nanoemulsion various extraction methods have been proposed including ultrafiltration, ultracentrifugation, gel filtration, and microdialysis. The drug incorporation efficiency is associated with its properties (i.e., lipophilicity, molecular weight, and structure).^69,102^ In order to determine the shelf life of the product the API content is evaluated in the nanoemulsions (previously tested for stability) using an appropriate analytical technique (i.e., HPLC or a spectrophotometric method).^30,66,85,87,92^

#### 5.1.12 Stability Study of Nanoemulsions

Nanoemulsions are thermodynamically unstable and undergo flocculation, coalescence, Ostwald maturation, and phase inversion during storage which may result in phase separation. Therefore, the final formulation of the nanoemulsion needs to remain both physically and chemically stable under ambient conditions during the production, storage, transport, and application. Alterations in the properties of nanoemulsions, resulting from the modification of pH, ionic strength, temperature, and mechanical forces, may lead to their destabilization and changes in particle size distribution and morphology which, in turn, may affect the release of substances from the dispersed phase.^63^

Stability assessment methods can either be based on the observation of emulsion systems over a specified period of time (emulsion aging method) or allow for a quick evaluation of the durability of the formulation (accelerated stability testing methods).^103^ The long-term stability study of nanoemulsions enables performing real-time stability evaluation as it does not use the conditions accelerating decomposition of the system.^103^

During long-term stability study, the formulations are stored at various specific temperatures for a period of 3–6 months, during which the properties of the nanoemulsion, that is, viscosity, pH, refractive index, average droplet size, and the content of the drug substance (see Table 5), are tested at different time points. Stable formulations are characterized by the lack of phase separation, a clear appearance, and only slight changes in physicochemical parameters.^100^

**Table 5:** Conditions for Assessing Long-Term Stability of Nanoemulsions

conditions	tested parameters	ref
*t* = 3 months	API content, mean size of the dispersed phase, pH, viscosity, refractive index	(100)
*T*_1_ = 4 °C		
*T*_2_ = 25 °C		
*T*_3_ = 37 °C		
*t* = 6 months	API content, mean size of dispersed phase, clarity, refractive index, viscosity, electrical conductivity, observation of phase separation	(85)
*T*_1_ = 4 °C		
*T*_2_ = 25 °C		
*T*_3_ = 40 °C		
stored away from light		
*t* = 3 months	pH, viscosity, mean size of the dispersed phase	(66)
*T*_1_ = 25 °C		
*T*_2_ = 40 °C		
stored in sealed bottles with a dropper		

Accelerated stability studies, that is, the centrifugal method and thermal tests, are also applied to assess the stability of nanoemulsions (Table 6). These methods can be utilized to accelerate the development of robust formulations during preformulation studies under a rigorous time frame.

**Table 6:** Examples of Accelerated Stability Studies of Nanoemulsions

method	conditions	ref
thermal	heating time: *t* = 48 h, *T* = 45 °C, no. of heating cycles: 6	(30)
	cooling time: *t* = 48 h, *T* = 4 °C, no. of cooling cycles: 6	
centrifugal	centrifugation: 3500 rpm, *t* = 30 min	
thermal	heating time: *t* = 48 h, *T* = 25 °C, no. of heating cycles: 3	
	freezing time: *t* = 48 h, *T* = −21 °C, no. of freezing cycles: 3	
thermal	freezing time: *t* = 24 h, *T* = −20 °C, no. of freezing cycles: 2–3	(96)
	heating time: *t* = 2–3 min, room temperature, no. of heating cycles: 2–3	
centrifugal	centrifugation: 5000 rpm, *t* = 30 min	
thermal	heating time: *t* = 24 h, *T* = 37 ± 0.5 °C, no. of heating cycles: 1	
	cooling time: *t* = 24 h, room temperature, no. of cooling cycles: 1	
centrifugal	centrifugation: 766*g*, *t* = 5 min, *T* = 25 °C, no. of cycles: 4	(66)

#### 5.1.14 Sterility Test

Sterility is one of the basic requirements for a formulation to be applied to the eyeball.^66^ The sterility test determines the presence of bacteria and fungi in a given preparation. In order to assess the sterility, the samples are inoculated under sterile conditions on microbiological media: a thioglycolate medium for the growth of aerobic or anaerobic bacteria and a medium with casein and soybean hydrolyzate for the growth of aerobic fungi and bacteria. According to the Ph. Eur. monograph (Ph. Eur. Chapter, Sterility: 2.6.1.) the samples should be incubated for 14 days at 30–35 °C in thioglycolate medium and at 20–25 °C in the casein and soybean hydrolyzate medium. If no microbial growth is observed in the samples, the tested product is considered to meet the sterility requirements.^16,104^

#### 5.1.15 *In Vitro* Drug Release from Nanoemulsions

The increased ability to solubilize the sparingly soluble active substance in nanoemulsions results in longer release of an API from these systems, as compared with conventional drug forms (e.g., eye drops), enabling the achievement of the therapeutic effect using a lower dose of the drug and to decrease the number of systemic side effects.^36^ The *in vitro* release study of the API from nanoemulsions allows determination of the release kinetics of the drug from a given formulation, which may provide preclinical data on the biodistribution and bioavailability of the drug into the eye. Sustained release formulations may provide the drug penetration into the deeper layers of the eye structure after application. Biorelevant methods of testing the release of ophthalmic products *in vitro* are still under development. Since there are no accepted compendial standards for this area we provide the overview of different noncompendial methods used for evaluation of drug release from ophthalmic nanoemulsions in this section. *In vitro* drug release from these systems is currently being assessed using a variety of membrane diffusion techniques including simple dialysis methods, dialysis methods using a modified type I or II apparatus, and Franz diffusion cells. The aforementioned USP type II apparatus is preferred for testing the release of substances from ophthalmic nanoemulsions. In this method, the formulation (0.5 mL) is placed in a dialysis bag and installed in the beaker containing an acceptor medium. The release test is usually performed at 34 ± 0.5 °C or 37 ± 0.5 °C in 900 mL of phosphate buffer at pH 7.4, often with addition of 1% sodium lauryl sulfate (SLS) or a buffered saline solution (PBS) at pH 7.4 with rotational speed of blades set to 50 rpm. The test is carried out in 3 replications for 6 h. The medium samples are withdrawn at specified intervals and the loss of the collected medium is replenished with a fresh buffer in order to maintain a constant fluid volume. The concentration of the active substance is determined using high-performance liquid chromatography (HPLC) or UV–vis spectroscopy.^29,30,40,85,86,96^

During the release of the API from nanoemulsion, the drug diffuses from the oil droplets into the surrounding aqueous environment. Depending on its solubility and the volume of the aqueous environment, the drug may dissolve or precipitate, which may lead to unreliable results. The method utilizing the type II apparatus can be used in the study of the release of substances from nanoemulsions when the concentration of the substance in the formulation exceeds its water solubility. Moreover, the large volume of the dissolution medium can help overcome the difficulties in maintaining sink conditions for poorly soluble drugs. *In vitro* drug release studies using membrane-free diffusion methods have also been described. However, because of the direct contact of the tested systems with the dissolution medium, their possible aggregation and/or disintegration in the dissolution medium should be assessed. *In vitro* drug release experiments are typically conducted under sink conditions which can be achieved with a relatively large volume of dissolution medium (i.e., from 40 to 200 mL). It can be expected that the small volume of the dissolution medium more accurately reflects the *in vivo* conditions since the average amount of tear fluid produced in the precorneal area during the 24 h period is 2 mL. However, in an *in vitro* drug release study from contact lenses, it has been shown that small volumes of dissolution medium are not suitable for *in vitro* testing or do not reflect the precorneal environment.

In order to minimize the effects of the unstirred aqueous layer, *in vitro* drug release experiments are performed at different agitation rates; for example, from 20 to 100 rpm for dialysis methods using a paddle dissolution apparatus, to 150 and 600 rpm for simple dialysis and Franz diffusion cell methods. Drug release studies are also performed using vertical Franz diffusion cells with an effective area of 1.13 cm^2^ into simulated tear fluid at pH 7.4. The nanoemulsion (1 mL) is deposited onto the previously soaked dialysis membrane which separates the donor and acceptor chambers, taking samples at regular intervals and replacing them with the same volume of fresh medium.

It should be stressed that sink conditions in the eye can be maintained if the drug clearance is high. However, the total clearance mechanism (including lacrimal turnover and absorption by the conjunctiva) is complex and difficult to simulate in *in vitro* studies. In physiological conditions the human eye contains a tear volume that ranges from 6.2 to 30.0 μL and the tear flow rate assumes values between 0.9 and 2.1 μL/min. In order to predict the drug release kinetics in the eye in a more reliable way , it is crucial to develop microfluidic models that mimic the hydrodynamic conditions of the eye as accurate as possible.

### 5.2 Determination of Drug Interactions with Other Components of Nanoemulsion

Understanding pharmaceutical nanoemulsions on a molecular level poses a significant analytical challenge because of the biphasic nature of the system (oil and water), relatively low concentration of API within formulations, and dynamics of the system in which drug and surfactant molecules can undergo continuous exchange between oil and water phase. Therefore, methods sensitive to changes in local and long-range structure and interactions within nanoemulsions needs to be applied. In this section we present examples of thermal and spectroscopic methods which can be employed to probe the interactions between functional components of nanoemulsions.

#### 5.2.1 Differential Scanning Calorimetry (DSC)

Differential scanning calorimetry is a thermoanalytical technique that determines the difference in the amount of heat delivered to the test sample compared to the reference. DSC is used to detect phase changes and the processes of crystallization of the oil phase and surfactants taking place in a nanoemulsion.^47,102^ Moghimipour et al. used DSC to investigate the changes in enthalpy and the phase transition temperature of bulk and bound water as a function of increasing oil content in the formulation. The statistical analysis proved that the increase of oil content and the surfactant/cosurfactant ratio significantly raise the enthalpy of the phase transition assigned to the so-called bound water freezing (at −17 to −26 °C), localized at the interface between the continuous phase (bulk water, at −8 to 0 °C) and the surfactants.^105^

#### 5.2.2 Nuclear Magnetic Resonance (NMR) and Fourier Transform Infrared (FTIR) spectroscopy

NMR spectroscopy is a powerful tool enabling the probe structure, dynamics, and interactions between constituents of a nanoemulsion with atomistic resolution (i.e., API, oil, surfactant, and cosurfactant). NMR data can be corroborated with FTIR spectra which is particularly sensitive to the changes in hydrogen bonding of the analyzed materials. Kumar et al. used a combination of NMR and FTIR spectroscopy to evaluate the interactions between an antifungal drug voriconazole and other components of the tested nanoemulsion. The acquired ^1^H NMR spectra of voriconazole and selected formulations did not present any significant shifts or broadening of the observed NMR peaks, indicating a lack of interaction between the drug and the nanoemulsion excipients. The NMR data was corroborated with FTIR spectra of the formulations which confirmed the presence of API vibrational bands.^86^ Chauham et al. compared the FITR spectra of pure diclofenac sodium salt to a formulation containing a drug-nanoemulsion system. The studies showed no significant differences in the position of the peaks in the obtained spectra, indicating a lack of interactions between the drug and the excipients.^106^

### 5.3 Biological Activity

#### 5.3.1 Evaluation of the Antifungal Activity of the Formulations

For nanoemulsions containing antifungal compounds the antifungal activity is evaluated using a solution of the analyzed drug as a reference. The evaluation of the developed carriers is performed by measuring the areas of growth inhibition of fungi, most often of the *Candida albicans* species, after application of the tested nanoemulsion formulations to agar plates containing the fungi and their incubation for 48 h at 25 °C.^80^

#### 5.3.2 Cytotoxicity Test

The determination of the cytotoxicity of the obtained nanoemulsions is of great importance to the safety of its application to the eyeball. Li et al. determined the cytotoxicity of a formulation against a fibroblast cell culture containing Earle’s balanced salt solution, 0.1 mM amino acids, 1.0 mM sodium pyruvate and 10% bovine serum solution, placed in an agar layer in a Petri dish. Macroscopical analysis after the incubation period resulted in either the absence or the appearance of a rim around the tested sample; in the case of latter its size was measured. The level of cytotoxicity was described using a 0–4 cytotoxicity scale. Within the applied scale grade 0 indicates no reactivity zone around or under the sample, grade 1 represents low reactivity - the zone diameter is limited to the area of the sample, grade 2 - mild reactivity - with the zone extending less than 0.5 cm the analyzed sample, grade 3 confirms moderate reactivity - with the measured zone extending 0.5–1.0 cm beyond the sample and grade 4 assign as severe reactivity - with the zone extending more than 1.0 cm beyond the sample.^92^

#### 5.3.3 Chicken Embryo Chorioallantoic Membrane Test - Hen’s Egg Test - Chorioallantoic Membrane (HET-CAM Test)

The HET-CAM test is an alternative test to the Draize eye irritation test, which enables the assessment of the toxicity level of the applied nanoemulsion formulations. As the chorioallantoic membrane (CAM) vascularization is resemblant to the mucosal tissue of humans, it is possible to use CAM to evaluate the irritating effect of tested materials and formulations *in vitro* prior to the *in vivo* and clinical tests.^17^ Freshly harvested fertilized hen eggs incubated for 3 days at 37 ± 0.5 °C are used for the test. In the fertilized eggs, a hole is made above the air chamber, through which the internal egg shell membrane (IESM) is moistened with a 0.9% sodium chloride solution. After a 20 min incubation the remaining physiological fluid and the IESM are removed revealing the chorioallantoic membrane. The test formulation is applied to the membrane, and the setup is monitored for any visible changes. Pathak et al. used a 0–3 scale (where 0 - nonirritated; 1 - mildly irritated; 2 - moderately irritated; 3 - severely irritated) to assess the toxicity of an ocular nanoemulsion in the HET-CAM test based on the degree of congestion, hemorrhage, and coagulation of the membrane blood vessels after the application of the sample.^80,107^

### 5.4 *Ex Vivo* Studies

#### 5.4.1 Assessment of Drug Substance Penetration through the Cornea

The study of drug substance penetration through the cornea is performed in the vertical Franz diffusion cell at 37 °C. Test formulation is placed in the donor part of the chamber and a freshly prepared artificial tear fluid solution at pH 7.4 or glutathione bicarbonate Ringer’s solution (GBR) is placed in the acceptor part of the diffusion cell. A cornea obtained from eyeballs of goats or albino rabbits is placed between the chambers. In order to assess the permeation of the drug substance, specific amounts of acceptor fluid are taken at time intervals, and the concentration of the tested substance is determined (e.g., using HPLC). Additionally, after the experiment, the changes to the epithelium can be evaluated under an optical microscope.^30,66,80^ Apart from the vertical Franz diffusion cell several other methods have been reported to date that can be used to evaluate *ex vivo* permeability of API formulated as nanoemulsions. The examples include: the modified Ussing chamber,^108^ the modified Franz diffusion cell,^109^ the horizontal perfusion cells,^110^ the modified Erlenmeyer flask diffusion cell,^111^ and the polycarbonate corneal perfusion chamber.^112^ After the *ex vivo* permeability study the apparent permeability coefficient (*P*_app_) is calculated using the following eq 1:^105,113^
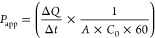
1where Δ*Q*/Δ*t* indicates the flux across the corneal tissue (μg·min^–1^) obtained from the slope of the linear portion of the permeation graph, 60 is the unit conversion from minutes to seconds, *A* is the corneal area available for penetration (1.7 cm^2^), and *C*_*0*_ is the initial concentration of the drug in the donor compartment.

#### 5.4.2 Assessment of Corneal Opacity

The corneal opacity test is another method enabling the assessment of toxicity of a drug substance. The test is performed on corneas of albino rabbits placed in the holder between the donor and acceptor chambers (the acceptor solution is usually a GBR buffer known as Ringer’s solution). In order to determine the degree of corneal opacity, the values of corneal absorbance before and after incubation with the tested nanoemulsion formulation are compared .^66^

### 5.5 *In Vivo* Studies

Too high ingredient concentration in a nanoemulsion may lead to ophthalmic tissues irritation as a result of an inappropriate emulsifier or oil selection. The tissue irritation can be manifested as excessive lacrimation, conjunctival hyperaemia, corneal swelling, or clouding. *In vivo* tissue scrutiny involves examination of the eye structures (i.e. the cornea, conjunctiva, and iris) after administration of the tested preparation. Despite the interspecies anatomical and physiological differences, such as the frequency of blinking or the permeability of the eye surface, albino rabbits (e.g., New Zealand rabbits) are most often used to assess the safety of a drug substance administered to the eye. This is due to the large corneal surface and conjunctival areas of the eye enabling easy observation of any changes that appear in the eye after application of the evaluated formulation. In addition, the iris of rabbits is pigment-free which allows for observation of the blood capillaries. The assessment of the safety profile of a nanoemulsion formulation after application can be performed using the Draize test or its modified versions (i.e., the Low Volume Eye Test (LVET test)).^114^

#### 5.5.1 Eye Irritation Test - Draize Eye Test, Low Volume Eye Test (LVET Test)

The Draize eye irritation test is traditionally performed to evaluate the irritation potential of pharmaceutical and cosmetic formulations in rabbit eyes. During the test 0.1 mL of the evaluated formulation is introduced into the conjunctival sac or administered directly to the rabbit’s cornea. Usually the left eyeball is treated as a control. In the modified Draize test (the LVET test) 0.01 mL of the evaluated formulation is introduced to the eye which reflects a realistic dose of the ocular formulations instilled to the eye in a single administration. The assessment of the changes in the eye is usually made 1, 24, 48, and 72 h after the application of the preparation and, if necessary, after 7 and 21 days. The condition of the eyeball is assessed through naked eye observation, using a magnifying glass or a slit lamp. The occurring changes are classified using the modified Friedenwald and Draize scale. Morsi et al. used a modified Draize scale (from 0 - no reaction to 4 - most severe reaction) to evaluate the cornea, iris, and conjunctiva irritation effect of an acetazolamide nanoemulsion. The observed changes include hemolysis, redness and inflammation. The evaluation score, expressed as the total eye irritation index, greater than 2 in each category indicated a strong irritant effect.^16,30,114,115^

#### 5.5.2 Histological Examination of the Eye Tissues

The histological examination of the rabbit eye is performed to assess the irritating effect and pathological changes that may appear after administration of the formulations. Fragments of eye tissues fixed in paraffin wax are evaluated. Swelling, bleeding, and other alterations in the epithelium of the retina, cornea, and ciliary body indicate an irritating effect of the formulation.^85^

#### 5.5.3 Assessment of Pharmacodynamic and Pharmacokinetic Parameters of Drugs Used in the Formulation in Comparison to the Reference Product

In order to evaluate the pharmacokinetic parameters: *C*_max_, *T*_max_ and AUC of an API in a form of nanoemulsion, a test formulation (or reference) is administered to the conjunctival sacs of rabbits, followed by sampling the aqueous humor from the anterior chamber of the eye and determination of the drug concentration by HPLC. This method was applied by Ligório Fialho and da Silva-Cunha to compare the pharmacokinetic parameters of dexamethasone microemulsion and the reference preparation.^100^ Morsi et al. assessed the pharmacokinetic parameters of the developed nanoemulsion formulations with acetazolamide: AUC, *T*_max_, mean residence time of the drug in the body (MRT), as well as maximum reduction of intraocular pressure (IOP) were compared with the control group receiving a reference formulation of brinzolamide. The experimental acetazolamide nanoemulsion or a reference formulation was administered to the 12 New Zealand rabbits with glaucoma (induced by injection of 0.25 mL of 2% sodium carboxymethylcellulose into the eye). At specified time intervals, the intraocular pressure was measured using a Schiötz tonometer which allowed for formulation comparison.^30^

#### 5.5.4 API Biodistribution into the Eye Compartments

To determine the biodistribution of a drug substance to different compartments of the eyeball and to the blood, Akhter et al. administered single drops of cyclosporin A nanoemulsions onto the eyeball of albino rabbits at specified time intervals. Then, in the collected biological samples (i.e., aqueous fluid, conjunctiva, cornea of the eyeball, and blood from the marginal ear vein), the authors determined the concentration of the API using the ultraperformance liquid chromatography method (UPLC) to assess the distribution of the substance to the aqueous fluid and the structures of the eye.^82,96^

### 5.6 Quality Control of Regulatory Importance

Quality control tests for ophthalmic formulations are based on pharmacopoeial standards and expanded by the internal product specifications that are of importance to ensure the product quality and determined by the manufacturer. These include the assessment of pH, tonicity, viscosity, clarity, compatibility with the eye, sterility, and others. The parameters should be tested rutinely not only as a part of batch assessment but also during the process (as intermediate stability data) and at the end of stability program. Furthermore, regulatory agencies tend to have additional expectations in terms of product specifications. Therefore, it is essential to work closely with regulatory agencies during formulation of novel drug delivery systems that must comply with regulatory requirements and guidelines. It is essential to identify critical quality attributes (CQAs) that are directly related to the product quality, efficacy, and toxicity, as they should form the basis for quality control of the product. CQAs for ophthalmic nanoemulsion may include aspects related not only to the technological process, such as particle size and size distribution, but also the purity and stability of API and key excipients after processing and during storage, as well as sterility and preservative efficiency for multidose products. Furthermore, the industrial quality control involves testing the final product that is nanoemulsion in a container. This implies additional tests that assess the integrity of the packing, withdrawal content, the interactions between the nanoemulsion components (API and excipients) with packing material as well as determination of a weight change upon storage. Examples of ophthalmic formulations quality tests with comments are given in Table 7.

**Table 7:** Examples of Quality Control Tests for Ophthalmic Formulations That Can Be Applied for Nanoemulsions^13,116,117^

quality control test	comments
potency/assay of active ingredient	The quantity of active pharmaceutical ingredient and possibly key excipients need to be determined. For nanoemulsions that may imply API content determination in both oil and water phases as well as the use and validation of advanced extraction protocols to assess the API content in the oil phase. The acceptable content of an API can be specified in the compendial monograph of the formulation (if available) or in general it is kept as 95% to 105% of declared content for initial analysis and 90% to 110% for shelf life stability analysis.
impurities	Qualitative and quantitative analysis of degradation products according to ICH guidelines. Impurity profile analysis using high-sensitivity analytical methods (e.g., mass spectrometry) is essential to determine the capability to detect a wide range of degradation products which may be generated across different stages of manufacturing (e.g., high-pressure processing or sterilization) during standard quality control process.
interactions	Determination of the interactions between the API and the container and critical excipients (e.g., preservatives, viscosity modifiers, antioxidants).
appearance (clarity, color, odor and consistency)	Discriminate a potential destabilization of the emulsions via creaming, coalescence, Ostwald ripening, and phase separation, changes caused by microorganisms or decomposition of nanoemulsion components (API or excipients).
particulates	The test determines the number of solid particles (in sizes above 10, 25, and 50 μm) per mL of the formulation. In case of ophthalmic nanoemulsions determination of this parameter should be possible with microscopic particle count test as light obscuration particle count test may be challenging due to colloidal nature of the formulation.
uniformity of dosage unit	The drug content delivered in each drop should be between 85% and 115% of the average drop content as determined using suitable, sensitive and validated method. Formulations that have large PDI and/or colloidal stability issues may not comply with this test as oil phase content may differ between droplets.
withdrawal content	The content withdrawn from the container must be not less than the label claim of the container. The tested product needs to comply with this requirement during the whole shelf life i.e. the content withdrawn at time zero needs to be equal the content withdrawn at the end of the shelf life of the product.
package integrity	Usually, visual appearance and function.
sterility	In compliance with pharmacopeial requirements.
preservative efficacy	Determines the level of antimicrobial activity of a product and can be related with the content of preservative in the formulation. The test enables to evaluate how well a product withstands microbial contamination during storage and use (this can be evaluated via in-use testing for multidose products).
stability studies	The stability program should be designed in accordance with ICH guidelines and in case of novel nanoemulsion based formulations should be discussed with and accepted by the regulatory body prior initiating to comply with the specific requirements.

## 6 Conclusion and Outlook

The ophthalmic drug market has significantly grown in recent years globally. Between 2015 and 2018 there was an 800% increase in approvals of new ophthalmic drugs including topical treatments.^118−120^ Although manufacturers are increasingly investing in research and development of innovative, noninvasive ophthalmic preparations, there is still a significant unmet need for the availability of multiactive ophthalmic drug formulations, including combination therapy products. Formulation of complex innovative drug delivery systems, such as ophthalmic nanoemulsions, requires extensive understanding of pharmaceutical aspects related to the selection of auxiliary substances and quality control methods that guarantee the desired properties, stability, and tolerance of the formulation during shelf life and after its application. Furthermore, from a formulation scientists’ perspective, it is of great importance to select robust optimizable technological processes enabling efficient manufacturing of ocular nanoemulsions on an industrial scale. This also includes formulation of sterile products as well as selection of packing and dosing devices.

Ophthalmic nanoemulsions formulations require optimization of both composition and manufacturing methods. The optimization process involves several critical nanoemulsion properties, for example, particle size and its distribution, stability, contact time at the application site, and tolerance. The available research indicates that size of oil phase droplets is strongly affected by the nanoemulsion composition, manufacturing technique, and preparation conditions. Therefore, there is a need for continuous research enabling to understand the interplay between the composition of nanoemulsions, their preparation techniques, and properties of the formulation. Furthermore, successful implementation of the nanoemulsion-based formulations into medical practice requires a comprehensive evaluation of their quality using well-defined, standardized analytical methods, and research protocols. Despite the continued interest in nanoemulsions formulation with low-energy methods, a growing share of the use of high-energy methods is observed. As low-energy methods do not require the use of expensive equipment, they are easy to implement in the laboratory and in preformulation studies. In addition, in the case of emulsification when low-energy methods are used, the composition of the formulation (e.g., surfactant to oil ratio, surfactants’ ratio in the mixture) primarily affects the properties of the final product as demonstrated by large number of studies on nanoemulsions obtained with low-energy methods. The growing share of research on the application of high-energy methods in the production of nanoemulsions is a consequence of their scalability and manufacturing capabilities of highly uniform batches important from the industrial perspective. In the case of emulsification using high-energy methods, both the composition and conditions of the emulsification process (e.g., device power level, pressure, temperature, homogenization time, or number of cycles) are important for the properties and stability of nanoemulsions. In further development of ophthalmic nanoemulsions formulation methods it is important to provide comparative studies on the critical parameters of products obtained using low- and high-energy methods. These would enable a better understanding of the influence of the formulation composition on manufacturing process as well as the advantages and limitations of both manufacturing approaches. In the case of high-energy manufacturing methods, it is advisible to assess the effect of each parameter on the quality of the product in order to determine the optimal process parameters’ range which, in turn, results in more reproducible and efficient production. This may translate into an increasing number of ocular nanoemulsions formulations available for patients which in accordance with an expected increasing demand for innovative ocular technologies.

## Supporting Information Available

Supporting tables: Table S1. Ophthalmic nanoemulsions formulations obtained using low-energy and high-energy methods; Table S2. Nanoemulsion eye drops in clinical trials; and Table S3. *In vitro*, *ex vivo*, and *in vivo* studies of the ophthalmic nanoemulsions formulations (PDF)
