# Supplementary material for: Ophthalmic Nanoemulsions: From Composition to Technological Processes and Quality Control
Source: Mol Pharm. 2021 Sep 17;18(10):3719–40. doi: 10.1021/acs.molpharmaceut.1c00650 (PMC8493553; doi:10.1021/acs.molpharmaceut.1c00650)
Supplement: Supplementary file 1 — mp1c00650_si_001.pdf [file mp1c00650_si_001.pdf]

Ophthalmic nanoemulsions: from composition to technological processes and quality control

Agnieszka Gawin-Mikołajewicz, Karol P. Nartowski*, Aleksandra J. Dyba, Anna M. Gołkowska, Katarzyna Malec, Bożena Karolewicz*

Department of Drug Form Technology, Wroclaw Medical University, Borowska 211 A, 50-556 Wroclaw, Poland

Table S1. Ophthalmic nanoemulsions formulations obtained using low-energy and high-energy methods.

API/ indication	Dispersed phase / Surfactants/co-surfactants / Continuous phase of nanoemulsion/ other components	Preparation method	Outcome	Ref.
Low-energy methods				
acetazolamide (1 wt. %) treatment of glaucoma	peanut oil, isopropyl myristate, oleic acid (3-6 wt. %)<br>Tween® 80, Kolliphor® EL/ Transcutol® P, propylene glycol (54-57 wt. %)<br>water with DMSO (dimethyl sulfoxide)/benzalkonium chloride (39wt. %)	Spontaneous emulsification	- peanut oil-based nanoemulsion showed better and prolonged intraocular hypotensive effect compared to commercially available brinzolamide eye drop (Azopt®) or available acetazolamide oral tablet (Cidamex®)	1
acyclovir (0.25 wt. %) treatment of viral eye infections	triacetin (5 wt. %)<br>Poloxamer® 407, Poloxamer® 188/Transcutol® P (12.5-30.0 wt. %)<br>water (69.75 – 87.25 wt. %)	Phase inversion composition method involving mixing of drug with oil and surfactants followed by addition of water and stirring for 3h	- in situ gelling nanoemulsion<br>- formulation with the composition of triacetin, Transcutol® P, Poloxamer® 407 and Poloxamer® 188 (inner phase droplet size 28 nm, polydispersity index 0.38), showed prolonged drug release compared to the control solution<br>- the drug penetration from the formulation was about 2.8 times higher than that of the control solution with good eye tolerance	2
atropine (0.5 wt. %) a therapeutic mydriatic	isopropyl myristate (10-25 wt. %)<br>Tween® 80 (50-65 wt. %), Span® 20 (50-65 wt. %)<br>Water (10-40 wt. %)	API was dissolved in a mixture of oil, surfactant and co-surfactant following dropwise addition of water under continuous stirring on magnetic stirrer	- nanoemulsions with oil droplets size below 50 nm was obtained<br>- the formulation with optimum properties (transparent, low viscosity - 20.1± 0.45 mPas) was composed of isopropyl myristate (10%), tween 80: span 20 (50%), and water (40%)	3

Table S1. (Continued) Ophthalmic nanoemulsions formulations obtained using low-energy and high-energy methods.

API/ indication	Dispersed phase	Surfactants/co-surfactants	Continuous phase of nanoemulsion/ other components	Preparation method	Outcome	Ref.
Low-energy methods						
brimonidine tartrate<br><br>treatment of glaucoma	castor oil, (1.87-5.67 wt. %)<br>Lipoid® S75, (0.5-1.0 wt. %)<br>Lipoid® E80, (0.25-0.50 wt. %)	Pluronic® F68, (0.75-1.5 wt. %)	methyl and<br>propyl paraben, water	Modified high shear homogenization method (combined phases were stirred with magnetic stirrer for 2h at 55°-65°C, following mechanical stirring for 30-45 min at 5,000-10,000 rpm at room temp. depending on the composition of nanoemulsion)	- inner phase droplets size of nanoemulsion < 30 nm, PDI < 0.35<br>- significant increase in therapeutic efficacy for various chronic ocular disease stages of both the anterior and posterior ocular segments	4
celecoxib<br>(1 wt. %)<br><br>treatment of age-related macular degeneration and diabetic retinopathy	oleic acid, Transcutol P® and mixture of oleic acid + Transcutol P [10:1],<br>(5 and 50 wt. %)	surfactants: Tween 80, Span 20, and cosurfactant propylene glycol, (40-90 wt. %)	water (5-10 wt. %)	Phase inversion composition method (the drug was dissolved in oil following addition of surfactant and cosurfactant mixture. Subsequently water was added drop wise under continuous stirring at ambient temperature)	- mean droplet size range of in the range of 6.96-26.65 nm<br>- 82.6% of the drug was released in 24h<br>- All NE formulations significantly increased partitioning, flux, and permeability coefficient of the drug through rabbit cornea	5
cyclosporine A<br>(0.01 - 1 wt. %)<br>dry eye syndrome (DES)	polyethoxylated castor oil or polyethoxylated hydrogenated castor oil – cyclosporine solubilizer (0.5 - 9.79 wt. %)		phosphate buffer<br>(90 - 99.29 wt. %)<br>thickening agent e.g. hyaluronic acid or its salt, glycerin, chitosan, cellulose derivatives, polyvinyl pyrrolidone (PVP), carboxy-methylcellulose (CMC), carbomer, polyethyleneoxide (PEO) (0.1 - 5 wt. %)<br>ethanol (0.1 - 3 wt. %)	Mixing the solution of cyclosporine A dissolved in a solubilizer; with a solution of thickening agent in phosphate buffer followed by stirring the resulting mixture without using a high speed stirring or shearing machine	- the effectiveness of the composition was confirmed based on the difference in the tears production between the right eye (induced dry eye group) and the left eye (dry eye syndrome group treated with the composition or Restasis®), increased tears production observed in the group treated with the tested combination 2, 3, 4 and 5 days after application<br>- nanoemulsions did not cause eye irritation or blurred vision after application<br>- the composition is stable during long-term storage period	6

Table S1. (Continued) Ophthalmic nanoemulsions formulations obtained using low-energy and high-energy methods.

API/ indication	Dispersed phase	Surfactants/co-surfactants	Continuous phase of nanoemulsion/ other components	Preparation method	Outcome	Ref.
Low-energy methods						
dorzolamide hydrochloride<br>(2.22 wt. %)<br>treatment of glaucoma	isopropyl myristate (2.00 wt. %), triacetin (glycerol triacetate) (2.00-4.50 wt. %)	Tween® 80 (6.75-13.50 wt.%), Kolliphor® EL (6.75-13.50 wt.%) / propylene glycol (4.00-4.50 wt. %), Transcutol® P (4.00-4.50 wt. %), Miranol® C2M Conc NP (4.50 wt. %)	Water (77.78 wt. %)/ benzalkonium chloride (0.01 wt. %)	Spontaneous emulsification using water titration method at 25 °C. The drug was added to the prepared nanoemulsions and vortexed until dissolved.	- the presence of Kolliphor® EL led to an increase in the bioavailability of the drug compared to the use of Tween® 80<br>- nanoemulsions were characterized by a rapid onset of action, prolonged drug release and increased bioavailability of the drug compared to the marketed product	7
fluconazole<br>(0.3 w/v)<br>treatment of fungal infections	Capmul® MCM (7 wt. %)	Transcutol® P (6.75 wt. %), Tween® 80 (20.25 wt. %)	water (ad 100 wt. %), carbopol 934 (0.1-0.5 w/v %)	Spontaneous emulsification using water titration method. The drug was added to the oil phase and vortexed until dissolved prior titration.	- the globule size in the range from 17.51-67.85 nm<br>- nanoemulsions formulated as in situ gelling system with longer residence time at the eye surface<br>- zeta potential strongly correlated with the addition of carbopol 934	8
moxifloxacin<br>(0.05% w/v)	ethyl oleate (4-20 wt. %)	Tween 80 and Soluphor P (S_{mix}, 4-20 wt. %)	water (44-60 wt. %), benzakonium chloride (0.005 wt. %)	API was dissolved in ethyl oleate and added to surfactant and co-surfactant mixture. The mixture was titrated with distilled water, followed by stirring and vortexing.	- droplet sizes below 81 nm<br>- the smallest droplets were obtained for the formulation with highest water content<br>- the nanoemulsions had adequate antimicrobial effect	9

Table S1. (Continued) Ophthalmic nanoemulsions formulations obtained using low-energy and high-energy methods.

API/ indication	Dispersed phase	Surfactants/co-surfactants	Continuous phase of nanoemulsion/ other components	Preparation method	Outcome	Ref.
Low-energy methods						
prostaglandins: latanoprost, travoprost, bimatoprost<br>(0.001-5.0 wt. %)<br><br>treatment of glaucoma	ethyl oleate, Miglyol® 812, castor oil (5.0-20.0 wt. %)	Tween® 80, Tween® 20, Brij® 58, Brij® 56, Brij® 52 (0.2-20.0 wt. %)	0.9% sodium chloride solution, phosphate buffer, citrate buffer/ chlorobutanol (50.0-95.0 wt. %)	Solubilizing surfactant and co-surfactant either in the aqueous or oily phase, following solubilisation of the API in the oily phase and mixing the oily phase with aqueous phase preferably with standard mixing procedures (e.g. paddle mixers, magnetic stirrers, homogenizers. High energy methods can be avoided.	- nanoemulsion with prostaglandin poorly soluble in water and unstable in aqueous solution, stabilized by the combination of two (or more) non-ionic surfactants<br>- a chemically and physically stable emulsion with a neutral zeta potential (-1.9 mV to 2.0 mV) was obtained<br>- the preparation was found to be non-toxic in both *in vitro* and *in vivo* tests	10
terbinafine hydrochloride<br>(0.5 wt. %)<br><br>treatment of fungal infections	isopropyl myristate (5-15 wt. %), Miglyol® 812 (5-15 wt. %)	Tween® 80, PEG 400, Kolliphor® EL (S_{mix}, 55 wt. %)	water (30-40 wt. %), benzalkonium chloride (0.01 wt. %)	Oil and water was mixed with surfactants mixture following vortexing.	- gellen gum was used to obtain in situ gelling formulation<br>- as compared to oil solution of the drug obtained formulation was non-irritant after application and improved the ocular drug bioavailability	11
travoprost<br>(0.004% w/v)<br><br>treatment of glaucoma	Labrafac Lipophile® oil (1-3% w/v)	Tween® 80 (0.5-2% w/v)	water (ad 100 mL)	API was mixed with oil using a magnetic stirrer. Surfactant was dissolved in water phase and added to the oil phase dropwise while stirring (500 rpm / 20 min.)	- particles size in the range from 157-291 nm<br>- formulations displayed extended release profile as compared with commercial product	12

Table S1. (Continued) Ophthalmic nanoemulsions formulations obtained using low-energy and high-energy methods.

API/ indication	Dispersed phase / Surfactants/co-surfactants / Continuous phase of nanoemulsion/ other components	Preparation method	Outcome	Ref.
High-energy methods				
alpha-2 adrenoceptor agonists (0.05-1 wt. %) dry eye syndrome (DES)	oils (1.25-5 wt. %): castor oil, olive oil, soybean oil, corn oil, mineral oil, cotton oil, safflower oil, sesame oil<br>Polysorbate® 80 (1-4 wt. %), poloxamer/tyloxapol (0.1-0.25 wt. %)<br>Water (q.s.) / carbomer (0.05 wt. %), glycerin (2.2 wt. %), sodium hydroxide (0.1 N q.s.), hydrochloric acid (q.s.), phosphate buffer (q.s), sodium citrate (0.1-0.2 wt. %), Tris base (0.03-0.15 wt. %), sodium EDTA (0.02 wt. %)	I. mixing with a shear mixer<br>II. microfluidization in several cycles, sterilization through a 0.22 µm filter / alternatively autoclaving at 121 °C for 20 min	- nanoemulsion without preservatives<br>- obtained inner phase droplets size < 200 nm	[13]
cyclosporine A, tacrolimus and sirolimus (0.2 wt. %) dry eye syndrome (DES)	castor oil (0.5-10. Wt. %)<br>Lipoid® E80 (0.1-2.0 wt. %), stearylamine (0.1-0.5 wt. %), Pluronic® F68 (0.5-2.0 wt. %),<br>Water (ad 100 wt. %) / vitamin E (0.01 wt. %), glycerol (2.25 wt. %), benzalkonium chloride (0.01 wt. %)	I. mixing with a magnetic stirrer (5 min)<br>II. mixing with a high-speed homogenizer (5 min)	- an emulsion forming a tear film that adheres electrostatically to the surface of the eye and moisturizes the eye<br>- nanoemulsion zeta potential value of 34-45 mV, average inner phase droplets size 150-250 nm	[14]
Dexamethasoneacetate / polymyxin B sulfate (0.05 wt. % / 0.1 wt. %) ocular inflammation such as chronic blepharitis and conjunctivitis	Eutanol® G:Lipoid® S 100 (70:30, 15-20 wt. %)<br>Tween® 80 (1-4 wt. %) Poloxamer® 407 (1-4 wt. %),<br>Preservative (cetylpyridinium chloride or benzalkonium chloride or cetalkonium chloride, 0.01 wt. %), glycerol (2.6 wt. %), sodium chloride (0.9 wt. %), water (ad 100 wt. %)	I. lipid phase was heated to 70 °C, followed by addition and dissolution of dexamethsone<br>II. the hot surfactant solution (70 °C) was added into the lipid phase under high speed stirring at 8000 rpm / min for 1 min<br>III. pre-emulsion was homogenized using high pressure homogenization (600 bar, 3 cycles, 70 °C)<br>IV. polymyxin B sulfate was admixed to the final nanoemulsions using continuous stirring at 200 rpm at RT	- inner phase droplets size <200 nm<br>- product stable within 180 days of storage at 4 °C and room temperature	[15]

Table S1. (Continued) Ophthalmic nanoemulsions formulations obtained using low-energy and high-energy methods.

API/ indication	Dispersed phase / Surfactants/co-surfactants / Continuous phase of nanoemulsion/ other components	Preparation method	Outcome	Ref.
High-energy methods				
ibuprofen<br><br>dry eye disease	Miglyol® 812 (5 wt. %), Lipoid® S 45 (0.1-1.0 wt. %)<br>Kolliphor® EL (0.25-2.5 wt. %)<br>Chitosan (0.05-0.5 wt. %), glycerol (2.5 wt. %), water (ad 100 wt. %)	I. mixing oil phase with lecithin following addition of aqueous phase and pre-homogenization with a shear mixer (5 min at 6,000 rpm)<br>II. the coarse emulsion was homogenized using microfluidization (1-15 cycles, 400- 1,300 bar) or high pressure homogenization (5 cycles, 1,000 bar)	- ibuprofen cationic nanoemulsion obtained by using microfluidization method with inner phase droplets size <200 nm, PDI < 0.3	16
immunosuppressant: cyclosporine, sirolimus or tacrolimus<br>(0.01-0.4 wt. %)<br>dry eye syndrome (DES)	medium-chain triglycerides (0.075-4 wt. %)<br>benzalkonium chloride (0.02 wt %), tyloxapol (0.12-0.3 wt. %), poloxamer (0.1 wt. %), vitamin E (0.01 wt. %)<br>Water (q.s.) / glycerin (2.25 wt. %)	I. oil and water phase components are mixed separately under slight heating with aid of magnetic stirring<br>II. aqueous phase is rapidly added to oily phase to form coarse emulsion, which is then heated to 75 °C and homogenized with high share mixer<br>III. final emulsion is obtained using several continuous cycles of microfluidization	- o/w emulsion with globule size < 250 nm and zeta potential in the range from 17.9 to 28.4 mV<br>- emulsion stable for 14 days during accelerated stability studies (at 80 °C)<br>-emulsion is well tolerated<br>- tissue concentrations of cyclosporine A were at least as good as the castor oil-based marketed emulsion Restasis®	17,18
lutein<br><br>supportive in prevention of age-related macular degeneration	isopropyl myristate (1-10.6 v. %), ethanol (4-42.4 v. %)<br>Tween® 80 (7.5-31.5 v. %), triacetin (2.5-10.5 v. %)<br>PBS (Phosphate Buffered Saline, 5-80 v. %)	I: the lutein was added to the mixture of oil and surfactant followed by stirring and sonication<br>II. the water phase was added to the oil phase under gentle stirring followed by ultrasonic homogenization and filtration through 0.45 µm PVDF filter	- for a period of 7 days after preparation no phase separation and particle size changes were observed<br>- an increased release of lutein and release prolongation was achieved<br>- it is a potential alternative to the available carriers for the lutein application	19

Table S1. (Continued) Ophthalmic nanoemulsions formulations obtained using low-energy and high-energy methods.

API/ indication	Dispersed phase / Surfactants/co-surfactants / Continuous phase of nanoemulsion/ other components	Preparation method	Outcome	Ref.
High-energy methods				
lutein (135 mg/L)<br><br>age-related macular degeneration (AMD)	MCT (1 wt%), Lipoid® E80 (1.8 wt. %), vitamin E (0.2 wt. %)<br>Poloxamer® 188 (0.1 wt. %)<br>water (q.s.)	I. mixing oil phase (MCT, phospholipids, vitamin E and ethyl acetate) at 60 °C and separately aqueous phase with poloxamer at 65 °C<br>II. mixing oil with aqueous phase with aid of high shear mixing followed by HPH (800 bar, 5 cycles)	- inner phase droplets size <110 nm, PDI < 0.2<br>- in situ gelling with sustained drug release up to 24 h	20
Latanoprost (0.005 wt. %), travoprost, bimatoprost, tafluprost, 8-iso-prostaglandin E2, isopropyl unoprostone<br><br>treatment of glaucoma	medium-chain triglycerides (1 wt. %)<br>ionic surfactant - benzalkonium chloride (0.02 wt. %), non-ionic surfactant - Lutrol® F68 (0.1-0.25 wt. %), tyloxapol (0.1-0.3 wt. %), vit E (0.005-0.01 wt. %)<br>water (ad 100 wt. %) / glycerin (2.25 wt. %)	I. API was dissolved in the oil phase with aid of heat,<br>II. Both oil and aqueous phases were heated to 65 °C, mixed together and heated to 75 °C rapidly and homogenized with high shear mixing (5 min.),<br>III. the obtained pre-emulsion was homogenized in a microfluidizer using 5-minute cycles under $6.89476x10^7$ Pa (10,000 psi).	- o/w cationic emulsion<br>- the emulsion droplets were in the range from 160 to 201 nm<br>- zeta potential was determined in the range from 20.6-22.4 mV<br>- latanoprost stability (80 °C, 14 days) in emulsion was improved as compared to commercial product	21

Table S1. (Continued) Ophthalmic nanoemulsions formulations obtained using low-energy and high-energy methods.

API/ indication	Dispersed phase	Surfactants/co-surfactants	Continuous phase of nanoemulsion/ other components	Preparation method	Outcome	Ref.
High-energy methods						
rifampicin<br>(0.1 wt. %)<br>eye infection caused by Mycobacterium tuberculosis	oleic acid (1.0 wt. %)	Poloxamer® 188 (0.7-0.9 wt. %),<br>Tween® 80 (0.7-0.9 wt. %)	water (ad. 100 wt. %) / chitosan (0.1-0.5 wt. %), polymyxin B (1000-5000 IU/mL)	I. oil and aqueous phases were heated separately at 70 ± 5 °C under continuous stirring (200 rpm / 30min, magnetic stirrer),<br>II. aqueous phase was added to oil phase followed by mixing with a magnetic stirrer (800 rpm / 1 min) and high-speed homogenizer (10,000 rpm / 5 min),<br>III. pre emulsion was homogenized using HPH (10,000 psi/ 5 cycles).	- obtained inner phase particle size about 150 nm, zeta potential value respectively + 51.3 mV (for the formulation with chitosan) and + 5.5 mV (for the formulation with polymyxin B)<br>- upon *in vitro* mucoadhesion evaluation of nanoemulsion the electrostatic interaction of cationic nanoemulsions with negatively charged mucin were observed<br>- nanoemulsion *in vitro* antibacterial activity evaluation showed that using the high pressure homogenization method and the surface modification process did not affect the antimicrobial efficacy of rifampicin<br>- obtained formulation with two substances i.e. polymyxin B and rifampicin revealed the possibility of simultaneous application of hydrophilic antibioticsthat may be important in the treatment of secondary and opportunistic bacterial infections accompanying with Mycobacterium tuberculosis eye infection	[22]
sirolimus<br><br>treatment of corneal inflammation in course of dry eye syndrome (DES)	medium-chain triglycerides (MCTs) (0.5-3 wt. %)	Lipoid® E80 (0.05-0.32 wt. %),<br>Lutrol® F86 (0.5 wt. %),<br>tyloxapol (0.16-0.32 wt. %),<br>Solutol® (0.2-0.32 wt. %)	water (ad 100 wt. %) / glycerol (2.25 wt. %), alpha tocopherol (0.01 wt. %), cetyltrimethylammonium bromide (CTAB) (0.05-0.1 wt. %), benzalkonium chloride (0.02-0.1 wt. %), oleylamine (0.1 w. %)	I. oil and aqueous phases were mixed were heated to 65 °C followed by mixing and rapid heating to 75 °C<br>II. obtained coarse emulsion was processed with high shear mixing (5 min)<br>III. the pre-emulsion was further homogenized with microfluidizer (5 min / 10,000 psi)	- o/w cationic nanoemulsion with a low concentration of cationic components (CTAB, benzalkonium chloride, oleylamine) and non-ionic surfactants (poloxamer, tyloxapol)<br>- obtained stable formulations with positive zeta potential, average inner phase droplets size 100–200 nm<br>- nanoemulsion with sirolimus in animal test was well tolerated by the rabbit eye	[23,24]

Table S1. (Continued) Ophthalmic nanoemulsions formulations obtained using low-energy and high-energy methods.

API/<br>indication	Dispersed phase<br>Surfactants/co-surfactants<br>Continuous phase of nanoemulsion/<br>other components	Preparation method	Outcome	Ref.
High-energy methods				
timolol<br><br>treatment of glaucoma	isopropyl myristate (5 wt. %)<br>Lipoid® E80 (1 wt. %), Tween® 80 (1.5 wt. %)<br>water (ad. 100 wt. %) / glycerol (2.5 wt. %), benzalkonium chloride (0.02 wt. %), EDTA (0.1 wt. %), polymers: chitosan, HEC, PVA, PEG, bis-(2-ethylhexyl) -sulfosuccinate sodium salt (AOT)	I. the oil and aqueous phases were mixed at 15, 000 rpm using rotational homogenizer,<br>II. the obtained pre-emulsion was homogenized using HPH (3 cycles, 5 min, 1,000 bar)	- stable, biocompatible, sterilizable and non-irritating nanoemulsions<br>- the use of polymers (chitosan, HEC, PVA, PEG) significantly increased the viscosity of the formulation<br>- formation of timolol ion pairs with AOT in selected formulations led to increased drug penetration through the cornea<br>- the presence of chitosan in nanoemulsions led to an increase in drug penetration	[25]
treatment of glaucoma (betaxolol), antibiotics, acyclovir, antiallergic drugs, anti-inflammatory agents (ibuprofen and its salts, diclofenac and its salts or indomethacin), antiviral drugs	oil phase comprises at least one oil having a molecular weight molecular mass of greater than 400 (2-40 wt. %)<br>fatty acid esters of sorbitan and ethoxylated sorbitan esters as surfactants, (0.2-15 wt. %)	I. mixing the water phase and the oil phase in the following conditions: temp. 10 - 80 °C, shearing 2×10^6 s⁻¹ - 5×10^8 s⁻¹<br>II. high pressure homogenizer, at least 1 cycle, pressure ranging from 6×10^7 Pa to 18×10^7 Pa	- the oil globules with an average size less than 100 nm<br>- the weight ratio between oily phase and surfactant is 2 to 10<br>- the emulsion has turbidity ranging from 60 to 600 NTU	[26]
treatment of glaucoma (betaxolol), antibiotics, acyclovir, antiallergic drugs, anti-inflammatory agents (ibuprofen and its salts, diclofenac and its salts or indomethacin), antiviral drugs	oil phase comprises at least one oil having a molecular weight molecular mass of greater than 400 (10-40 wt. %)<br>ethoxylated fatty ethers and ethoxylated fatty esters, and at least one oil, (0.2-15 wt. %)<br>Water (ad 100 wt. %) / glycerin (5 wt. %)	I. mixing the water phase and the oil phase in the following conditions: temp.: 10 - 80 °C, shear rate: 2×10^6 s⁻¹ - 5×10^8 s⁻¹<br>II. high pressure homogenizer, at least 1 cycle, pressure ranging from 6×10^7 Pa to 18×10^7 Pa	- the oil globules with an average size less than 100 nm<br>- the weight ratio between oily phase and surfactant is 2 to 10<br>- the emulsion has turbidity ranging from 60 to 600 NTU	[27]

Table S1. (Continued) Ophthalmic nanoemulsions formulations obtained using low-energy and high-energy methods.

<table>
<tr><td rowspan="3">API/
indication</td><td>Dispersed phase</td><td rowspan="3">Preparation method</td><td rowspan="3">Outcome</td><td rowspan="3">Ref.</td></tr>
<tr><td>Surfactants/co-surfactants</td></tr>
<tr><td>Continuous phase of nanoemulsion/
other components</td></tr>
<tr><td colspan="5">High-energy methods</td></tr>
<tr><td rowspan="3">treatment of glaucoma (betaxolol), antibiotics, acyclovir, antiallergic drugs, anti-inflammatory agents (ibuprofen and its salts, diclofenac and its salts or indomethacin), antiviral drugs</td><td>PEG 400 isostearate (4.5 wt. %), disodium acylglutamate (0.5 wt. %), isopropyl myristate (5 wt. %), isocetyl stearate (10 wt. %)</td><td rowspan="3">I. mixing the water phase and the oil phase at an ambient temperature below 45 °C, with a shearing rate in the range from $2x10^6$ s^{-1} to $5x10^8$ s^{-1}
II. high pressure homogenization preferably at pressure in the range from $6x10^7$ Pa to $18x10^7$ Pa</td><td rowspan="3">- the oil globules with an average size less than 100 nm, viscosity ranging from 1-200 Poises measured at 25 °C at 200 s^{-1}
- o/w nanoemulsions containing non-ionic polymers or mixtures of polymers and homopolymers
- transparent, stable nanoemulsions with the oil phase particles size <100 nm were obtained
- the viscosity of the nanoemulsion increased ca. 5-fold when a polymer concentration of 1 wt. % was used</td><td rowspan="3">[28]</td></tr>
<tr><td></td></tr>
<tr><td>water (ad 100 wt. %) / glycerol (5 wt. %), dipropylene glycol (10 wt. %), hydroxypropyl guar (0.8 wt. %), poly(ethylene oxide) (300,000 g/mol, 3 wt. %), Natrosol® 250HHR (hydroxyl-ethylcellulose, 0.5 wt. %), Carbopol® 980 (0.26 wt. %), triethanolamine (0.39 wt. %)</td></tr>
</table>

Table S2. Nanoemulsion eye drops in clinical trials.

Substance	Condition or disease	Phase and type of study	Trial Identifier	Sponsor/Country/Study date
Brimonidine tartrate (0,18%)	Graft versus Host Disease (oGVHD)	Phase 3, study randomized, placebo-controlled, double-masked clinical trial	ClinicalTrials.gov Identifier: NCT03591874 [29]	Ocugen/United States/2018 – 2020
Brimonidine tartrate (0,20%)	Dry eye disease	Phase 3, study randomized, placebo-controlled, double-masked clinical trial	ClinicalTrials.gov Identifier: NCT03785340[30]	Ocugen/United States/2018 – 2019
Clobetasol propionate (0,05%)	Treatment of inflammation and pain associated with cataract surgery	Phase 3, study randomized, placebo-controlled, double-masked clinical trial	ClinicalTrials.gov Identifier: NCT04246801[31] ClinicalTrials.gov Identifier: NCT04249076[32]	Salvat/United States/2020 - present
Propylene glycol-based eye drops	Dry eye disease	Evaluation of the Clinical Efficacy and Tolerability of SYSTANE Complete in Adult Patients With Dry Eye Disease Following Topical Ocular Use for 4 Weeks: A Multicenter Trial	ClinicalTrials.gov Identifier: NCT03492541[33]	Alcon Research/United States/2018 – 2019
Ikervis® (1mg/mL Ciclosporin)	Keratitis-ichthyosis-deafness syndrome	Phase 3, study randomized, double-masked clinical trial, 3-year Study to Explore the Long-term Evolution of Sign and Symptoms, and Occurence of Complications in Dry Eye Patients With Severe Keratitis Receiving	ClinicalTrials.gov Identifier: NCT04144413[34]	Santen SAS/United States/2019 - present
Ikervis® (1mg/mL Ciclosporin)	Moderate to Severe Dry Eye	Phase 4	ClinicalTrials.gov Identifier: NCT04775303[35]	Yonsei University/Republic of Korea/2020 - present
Ikervis® (1mg/mL Ciclosporin)	Prophylaxis od dry eye disease in leukemia (Both ALL and AML) and myelodysplastic syndrome with excess blast (MDS-EB-1)	Phase 4	ClinicalTrials.gov Identifier: NCT04636918[36]	Singapore Eye Research Institute/Singapore/2019 - present
Ikervis® (1mg/mL Ciclosporin) vs cationorm eye drops	Ocular Surface Disease Ocular Inflammation	Phase 1	ClinicalTrials.gov Identifier: NCT04812951[37]	Singapore Eye Research Institute/Singapore/2021 - present
Restasis (Cyclosporine Ophthalmic Emulsion 0.05%)	Ocular Surface Disease	Phase 1, Phase 2 Phase 3	ClinicalTrials.gov Identifier: NCT04918823[38] ClinicalTrials.gov Identifier: NCT04735510[39]	Campus Bio-Medico University/Italy/2021 – present Boston Sight/United States/2021-present

Table S3. In vitro, ex vivo and in vivo studies of the ophthalmic nanoemulsions formulations.

Study type	Used equipment/test conditions	API	Ref.
In vitro studies			
Visual assessment and the transmittance percentage	UV-Vis spectrophotometer	atropine	[3]
		fluconazole	[8]
	photoelectric colorimeter	moxifloxacin	[9]
Morphological characteristics and droplet size analysis of the dispersed phase	dynamic light scattering / photon correlation spectroscopy / quasi-elastic light scattering	acetazolamide	[1]
		celecoxib	[5]
		curcumin	[40]
		cyclosporine A	[41]
		dexamethasone acetate, polymyxin B	[15]
		diclofenac sodium	[42]
		dorzolamide hydrochloride	[7]
		fluconazole	[8]
		ibuprofen	[16]
		lutein	[19,20]
		moxifloxacin	[9]
		pilocarpine hydrochloride	[43]
		terbinafine hydrochloride	[11]
		timolol maleate	[25]
		travoprost	[12]
		voriconazole	[44]
	microscopic methods (optical microscope, polarizing microscope, atomic force microscope - AFM, transmission electron microscope - TEM, scanning electron microscope - SEM)	acetazolamide	[1]
		atropine	[3]
		curcumin	[40]
		cyclosporin A	[41]
		diclofenac sodium	[42]
		fluconazole	[8]
		ibuprofen	[16]
		lutein	[19]
		moxifloxacin	[9]
		terbinafine hydrochloride	[11]
		travoprost	[12]
		voriconazole	[44]

Table S3. (Continued) In vitro, ex vivo and in vivo studies of the ophthalmic nanoemulsions formulations.

Study type	Used equipment/test conditions	API	Ref.
In vitro studies			
Zeta potential measurement	dynamic light scattering	cyclosporine A	41
		dexamethasone acetate, polymyxin B	15
		diclofenac sodium	42
		fluconazole	8
		ibuprofen	16
		moxifloxacin	9
		timolol maleate	25
		travoprost	12
pH measurement	pH meter	acetazolamide	1
		atropine	3
		celecoxib	5
		cyclosporine A	41
		dexamethasone acetate, polymyxin B	15
		dorzolamide hydrochloride	7
		fluconazole	8
		ibuprofen	16
		moxifloxacin	9
		travoprost	12
		voriconazole	44
Refractive index measurement	refractometer<br>e.g. Abbe refractometer	acetazolamide	1
		atropine	3
		curcumin	40
		cyclosporine A	41
		dorzolamide hydrochloride	7
		pilocarpine hydrochloride	43
		travoprost	12
Osmolarity measurement	osmometer	acetazolamide	1
		dexamethasone acetate, polymyxin B	15
		dorzolamide hydrochloride	7
		ibuprofen	16
		timolol maleate	25

Table S3. (Continued) In vitro, ex vivo and in vivo studies of the ophthalmic nanoemulsions formulations.

Study type	Used equipment/test conditions	API	Ref.
In vitro studies			
Conductivity	conductometer	atropine	[3]
		moxifloxacin	[9]
		pilocarpine hydrochloride	[43]
Surface tension measurement	tensiometer	acetazolamide	[1]
		dorzolamide hydrochloride	[7]
		ibuprofen	[16]
		timolol maleate	[25]
Viscosity determination	viscometer, rheometer	acetazolamide	[1]
		atropine	[3]
		celecoxib	[5]
		curcumin	[40]
		cyclosporine A	[41]
		dexamethasone	[45]
		dexamethasone acetate, polymyxin B	[15]
		diclofenac sodium	[42]
		dorzolamide hydrochloride	[7]
		fluconazole	[8]
		lutein	[19]
		moxifloxacin	[9]
		pilocarpine hydrochloride	[43]
		terbinafine hydrochloride	[11]
		timolol maleate	[25]
		voriconazole	[44]
Drug content determination	HPLC , UPLC	curcumin	[40]
		cyclosporine A	[41]
		ibuprofen	[16]
		lutein	[19]
		moxifloxacin	[9]
		pilocarpine hydrochloride	[43]
		timolol maleate	[25]
		travoprost	[12]
		voriconazole	[44]
	UV-Vis spectrophotometer	acetazolamide	[1]
		celecoxib	[5]
		diclofenac sodium	[42]

Table S3. (Continued) In vitro, ex vivo and in vivo studies of the ophthalmic nanoemulsions formulations.

Study type	Used equipment/test conditions	API	Ref.
In vitro studies			
Stability studies	accelerated stability test	acetazolamide	[1]
		celecoxib	[5]
		curcumin	[40]
		cyclosporine A	[41]
		dorzolamide hydrochloride	[7]
		fluconazole	[8]
		ibuprofen	[16]
		moxifloxacin	[9]
		terbinafine hydrochloride	[11]
		timolol maleate	[25]
		travoprost	[12]
		voriconazole	[44]
	long-term stability test	celecoxib	[5]
		dexamethasone	[45]
		dexamethasone acetate, polymyxin B	[15]
		ibuprofen	[16]
		moxifloxacin	[9]
		pilocarpine hydrochloride	[43]
		timolol maleate	[25]
Mucoadhesion measurement	mucin solutions	dexamethasone acetate, polymyxin B	[15]
		ibuprofen	[16]
	texture analysis	cyclosporine A	[41]
	modified balance method	acetazolamide	[1]
	assessment of the 'rheological synergism'	terbinafine hydrochloride	[11]
Sterility test	involving fluid thioglycolate medium and soybean-casein digest medium	clotrimazole	[46]

Table S3. (Continued) In vitro, ex vivo and in vivo studies of the ophthalmic nanoemulsions formulations.

Study type	Used equipment/test conditions	API	Ref.
In vitro studies			
In vitro study of drug release	USP II apparatus for drug release testing, semi-permeable membrane	acetazolamide	[1]
		dorzolamide hydrochloride	[7]
		ibuprofen	[16]
		pilocarpine hydrochloride	[43]
		terbinafine hydrochloride	[11]
		travoprost	[12]
		voriconazole	[44]
	Franz diffusion cell	celecoxib	[5]
		cyclosporine A	[41]
		diclofenac sodium	[42]
		moxifloxacin	[9]
Studies of drug / formulation components interactions	Nuclear Magnetic Resonance Spectrometer (NMR spectrometer)	voriconazole	[44]
	Fourier-transform infrared spectrometer (FTIR spectrometer)	diclofenac sodium salt	[42]
	Differential Scanning Calorimeter (DSC)	celecoxib	[5]
Evaluation of antifungal activity	measurement of Candida albicans growth inhibition areas diameters	fluconazole	[8]
Evaluation of antimicrobial activity	agar diffusion test	moxifloxacin	[9]
Cytotoxicity test	fibroblast cell culture	dexamethasone acetate, polymyxin B	[15]
	human corneal epithelial cells	ibuprofen	[16]
HET-CAM test	chorioallantoic membrane	fluconazole	[8]
Ex vivo studies			
Assessment of drug penetration through the cornea	Franz diffusion cell, other diffusion cell	acetazolamide	[1]
		celecoxib	[5]
		diclofenac sodium	[42]
		fluconazole	[8]
		moxifloxacin	[9]
		timolol maleate	[25]
		voriconazole	[44]
Assessing corneal opacity	measuring the absorbance values	timolol maleate	[25]

Table S3. (Continued) In vitro, ex vivo and in vivo studies of the ophthalmic nanoemulsions formulations.

Study type	Used equipment/test conditions	API	Ref.
In vivo studies			
Eye irritation assessment: Draize rabbit eye test, Low Volume Eye Test (LVET test)	scoring on modified Friedenwald's and Draize's grading system	acetazolamide	1
		cyclosporin A	41
		dorzolamide hydrochloride	7
		moxifloxacin	9
		travoprost	12
Histological examination of an eye	optical microscope	dorzolamide hydrochloride	7
		fluconazole	8
		lutein	20
		pilocarpine hydrochloride	43
		terbinafine hydrochloride	11
		travoprost	12
Assessment of pharmacodynamic and pharmacokinetic parameters	determination of the drug concentration in the eye structures (HPLC, UPLC)	cyclosporine A	41
		dexamethasone	45
		moxifloxacin	9
		terbinafine hydrochloride	11
		travoprost	12
	intraocular pressure measurement, Schiötz tonometer	acetazolamide	1
		dorzolamide hydrochloride	7
		pilocarpine hydrochloride	43
		travoprost	12

References:

(1) Morsi, N. M.; Mohamed, M. I.; Refai, H.; El Sorogy, H. M. Nanoemulsion as a Novel Ophthalmic Delivery System for Acetazolamide. *Int. J. Pharm. Pharm. Sci.* **2014**, *6* (11), 227–236.

(2) Mahboobian, M. M.; Mohammadi, M.; Mansouri, Z. Development of Thermosensitive in Situ Gel Nanoemulsions for Ocular Delivery of Acyclovir. *J. Drug Deliv. Sci. Technol.* **2020**, *55*, 101400. https://doi.org/10.1016/j.jddst.2019.101400.

(3) Badmapriya, D.; Rajalakshmi, A. N. Formulation and Evaluation of Atropine Microemulsion as Ocular Drug Delivery. *Int. J. Pharmacy&Technology IJPT* **2010**, *2* (4), 924–931.

(4) Rimple; Newton, M. J. Impact of Ocular Compatible Lipoids and Castor Oil in Fabrication of Brimonidine Tartrate Nanoemulsions by 3 3 Full Factorial Design. *Recent Pat. Inflamm. Allergy Drug Discov.* **2018**, *12* (2), 169–183. https://doi.org/10.2174/1872213x12666180730115225.

(5) Moghimipour, E.; Salimi, A.; Yousefvand, T. Preparation and Evaluation of Celecoxib Nanoemulsion for Ocular Drug Delivery. *Asian J. Pharm.* **2017**, *11* (3), 543–550.

(6) Hwang, S. J.; Cha, K. H.; Kang, H.; Sun, B. K. Cyclosporine-Containing, Non-Irritative Nanoemulsion

Opthalmic Composition. EP2845602B1, 2017.

(7) Ammar, H. O.; Salama, H. A.; Ghorab, M.; Mahmoud, A. A. Nanoemulsion as a Potential Ophthalmic Delivery System for Dorzolamide Hydrochloride. *AAPS PharmSciTech* **2009**, *10* (3), 808–819. https://doi.org/10.1208/s12249-009-9268-4.

(8) Pathak, M. K.; Chhabra, G.; Pathak, K. Design and Development of a Novel PH Triggered Nanoemulsified In-Situ Ophthalmic Gel of Fluconazole: Ex-Vivo Transcorneal Permeation, Corneal Toxicity and Irritation Testing. *Drug Dev. Ind. Pharm.* **2013**, *39* (5), 780–790. https://doi.org/10.3109/03639045.2012.707203.

(9) Shah, J.; Nair, A. B.; Jacob, S.; Patel, R. K.; Shah, H.; Shehata, T. M.; Morsy, M. A. Nanoemulsion Based Vehicle for Effective Ocular Delivery of Moxifloxacin Using Experimental Design and Pharmacokinetic Study in Rabbits. *Pharmaceutics* **2019**, *11* (5). https://doi.org/10.3390/pharmaceutics11050230.

(10) Carli, F.; Mihran, B.; Schmid, R.; Chiellini, E. Ophthalmic Oil-in-Water Emulsions Containing Prostaglandins. US8414904B2, 2013.

(11) Tayel, S. A.; El-Nabarawi, M. A.; Tadros, M. I.; Abd-Elsalam, W. H. Promising Ion-Sensitive in Situ Ocular Nanoemulsion Gels of Terbinafine Hydrochloride: Design, in Vitro Characterization and in Vivo Estimation of the Ocular Irritation and Drug Pharmacokinetics in the Aqueous Humor of Rabbits. *Int. J. Pharm.* **2013**, *443* (1–2), 293–305. https://doi.org/10.1016/j.ijpharm.2012.12.049.

(12) Ismail, A.; Nasr, M.; Sammour, O. Nanoemulsion as a Feasible and Biocompatible Carrier for Ocular Delivery of Travoprost: Improved Pharmacokinetic/Pharmacodynamic Properties. *Int. J. Pharm.* **2020**, *583*, 119402. https://doi.org/10.1016/j.ijpharm.2020.119402.

(13) Jain, S.; Kompella, U. B.; Musunuri, S. Preservative Free Ocular Compositions and Methods for Using the Same for Treating Dry Eye Disease and Other Eye Disorders. US20180153885A1, 2020.

(14) Benita, S.; Lambert, G. Method and Composition for Dry Eye Treatment. US6656460B2, 2003.

(15) Li, X.; Muller, R. H.; Keck, C. M.; Bou-Chacra, N. A. Mucoadhesive Dexamethasone Acetate-Polymyxin B Sulfate Cationic Ocular Nanoemulsion - Novel Combinatorial Formulation Concept. *Pharmazie* **2016**, *71* (6), 327–333. https://doi.org/10.1691/ph.2016.5190.

(16) Jurišić Dukovski, B.; Juretić, M.; Bračko, D.; Randjelović, D.; Savić, S.; Crespo Moral, M.; Diebold, Y.; Filipović-Grčić, J.; Pepić, I.; Lovrić, J. Functional Ibuprofen-Loaded Cationic Nanoemulsion: Development and Optimization for Dry Eye Disease Treatment. *Int. J. Pharm.* **2020**, *576*, 118979. https://doi.org/10.1016/j.ijpharm.2019.118979.

(17) Philips, B.; Bague, S.; Rabinovich-Guilatt, L.; Lambert, G. Ophthalmic Emulsions Containing an Immunosuppressive Agent. EP1809238B1, 2008.

(18) Philips, B.; Bague, S.; Rabinovich-Guilatt, L.; Lambert, G. Ophthalmic Emulsions Containing an Immunosuppressive Agent. US8298569B2, 2012.

(19) Lim, C.; Kim, D. won; Sim, T.; Hoang, N. H.; Lee, J. W.; Lee, E. S.; Youn, Y. S.; Oh, K. T. Preparation and Characterization of a Lutein Loading Nanoemulsion System for Ophthalmic Eye Drops. *J. Drug Deliv. Sci. Technol.* **2016**, *36*, 168–174. https://doi.org/10.1016/j.jddst.2016.10.009.

(20) Ge, Y.; Zhang, A.; Sun, R.; Xu, J.; Yin, T.; He, H.; Gou, J.; Kong, J.; Zhang, Y.; Tang, X. Penetratin-Modified Lutein Nanoemulsion in-Situ Gel for the Treatment of Age-Related Macular Degeneration. *Expert Opin. Drug Deliv.* **2020**, *17* (4), 603–619. https://doi.org/10.1080/17425247.2020.1735348.

(21) Philips, B.; Bague, S.; Rabinovich-Guilatt, L.; Lambert, G. Ophthalmic Emulsions Containing Prostaglandins. EP1827373B1, 2008.

(22) Bazán Henostroza, M. A.; Curo Melo, K. J.; Nishitani Yukuyama, M.; Löbenberg, R.; Araci Bou-Chacra, N. Cationic Rifampicin Nanoemulsion for the Treatment of Ocular Tuberculosis. *Colloids Surfaces A Physicochem. Eng. Asp.* **2020**, *597*, 124755. https://doi.org/10.1016/j.colsurfa.2020.124755.

(23) Bague, S.; Philips, B.; Garrigue, J.-S.; Rabinovich-Guilatt, L.; Lambert, G. Oil-in-Water Type Emulsion with Low Concentration of Cationic Agent and Positive Zeta Potential. US8298568B2, 2012.

(24) Bague, S.; Philips, B.; Garrigue, J.-S.; Rabinovich-Guilatt, L.; Lambert, G. Oil-in-Water Type Emulsion with Low Concentration of Cationic Agent and Positive Zeta Potential. EP 1655021 B1, 2008.

(25) Gallarate, M.; Chirio, D.; Bussano, R.; Peira, E.; Battaglia, L.; Baratta, F.; Trotta, M. Development of O/W Nanoemulsions for Ophthalmic Administration of Timolol. *Int. J. Pharm.* **2013**, *440* (2), 126–134. https://doi.org/10.1016/j.ijpharm.2012.10.015.

(26) Simonnet, J.-T.; Sonneville, O.; Legret, S. Nanoemulsion Based on Oxyethylenated or Non-Oxyethylenated Sorbitan Fatty Esters, and Its Uses in the Cosmetics, Dermatological and/or Ophthalmological Fields. US6335022B1, 2002.

(27) Simonnet, J.-T.; Sonneville, O.; Legret, S. Nanoemulsion Based on Ethoxylated Fatty Ethers or on Ethoxylated Fatty Esters and Its Uses in the Cosmetics, Dermatological and/or Ophthalmological Fields. US6375960B1, 2002.

(28) L'Alloret, F.; Aubrun-Sonneville, O.; Simonnet, J.-T. Nanoemulsion Containing Nonionic Polymers, and Its Uses. US6998426B2, 2006.

(29) Study of Brimonidine Tartrate Nanoemulsion Eye Drops in Patients With Ocular Graft-vs-Host Disease - Full Text View - ClinicalTrials.gov https://clinicaltrials.gov/ct2/show/NCT03591874 (accessed Apr 29, 2021).

(30) Study of Brimonidine Tartrate Nanoemulsion Eye Drop Solution in the Treatment of Dry Eye Disease (DED) - Full Text View - ClinicalTrials.gov https://clinicaltrials.gov/ct2/show/NCT03785340 (accessed Apr 29, 2021).

(31) Clobetasol Propionate Ophthalmic Nanoemulsion 0.05% for the Treatment of Inflammation and Pain Associated With Cataract Surgery (CLOSE-1) - Full Text View - ClinicalTrials.gov https://clinicaltrials.gov/ct2/show/NCT04246801 (accessed Apr 29, 2021).

(32) Clobetasol Propionate Ophthalmic Nanoemulsion 0.05% for the Treatment of Inflammation and Pain Associated With Cataract Surgery (CLOSE-2) - Full Text View - ClinicalTrials.gov https://clinicaltrials.gov/ct2/show/NCT04249076 (accessed Apr 29, 2021).

(33) Study of Efficacy and Tolerability of SYSTANE Complete in Patients With Dry Eye Disease - Full Text View - ClinicalTrials.gov https://clinicaltrials.gov/ct2/show/NCT03492541 (accessed Jul 22, 2021).

(34) 3-year Study in Dry Eye Disease Patients With Severe Keratitis Receiving Ikervis® (1mg/mL Ciclosporin) - Full Text View - ClinicalTrials.gov https://clinicaltrials.gov/ct2/show/NCT04144413 (accessed Jul 22, 2021).

(35) A Clinical Trial to Evaluate the Clinical Efficacy of Cyclosporine 0.1% (Ikervis®) for Moderate to Severe Dry Eye Patients. (IKE-03-SWITCHING) - Full Text View - ClinicalTrials.gov https://clinicaltrials.gov/ct2/show/NCT04775303 (accessed Jul 22, 2021).

(36) Ikervis for DED Due to GVHD Post Allo-HSCT - Full Text View - ClinicalTrials.gov https://clinicaltrials.gov/ct2/show/NCT04636918 (accessed Jul 22, 2021).

(37) Cyclosporine 0.1% Eye Drops as Prophylactic Treatment In Cataract Surgery - Full Text View - ClinicalTrials.gov https://clinicaltrials.gov/ct2/show/NCT04812951 (accessed Jul 22, 2021).

(38) Novel Use of Cyclosporine Ophthalmic Emulsion 0.05% on Application of PROSE Devices for Management of Patients With Ocular Surface Disease - Full Text View - ClinicalTrials.gov https://clinicaltrials.gov/ct2/show/NCT04918823 (accessed Jul 22, 2021).

(39) Novel Use of Restasis and PROSE Devices - Full Text View - ClinicalTrials.gov https://clinicaltrials.gov/ct2/show/NCT04735510 (accessed Jul 22, 2021).

(40) Anjana, D.; Anitha Nair, K.; Somashekara, N.; Venkata, M.; Sripathy, R.; Yelucheri, R.; Parmar, H.; Upadhyay, R.; Rama Verma, S.; Ramchand, C. N. Development of Curcumin Based Ophthalmic

Formulation. *Am. J. Infect. Dis.* **2012**, *8* (1), 41–49. https://doi.org/10.3844/ajidsp.2012.41.49.

(41) Akhter, S.; Anwar, M.; Siddiqui, M. A.; Ahmad, I.; Ahmad, J.; Ahmad, M. Z.; Bhatnagar, A.; Ahmad, F. J. Improving the Topical Ocular Pharmacokinetics of an Immunosuppressant Agent with Mucoadhesive Nanoemulsions: Formulation Development, in-Vitro and in-Vivo Studies. *Colloids Surfaces B Biointerfaces* **2016**, *148*, 19–29. https://doi.org/10.1016/j.colsurfb.2016.08.048.

(42) Chauhan, S.; Road, M. Development and In Vitro Characterization of Nanoemulsion Embedded Thermosensitive In-Situ Ocular Gel of Diclofenac Sodium for Sustained Delivery. *Int. J. Pharm. Sci. Res.* **2018**, *9* (6), 2301–2314. https://doi.org/10.13040/IJPSR.0975-8232.9(6).2301-14.

(43) Ince, I.; Karasulu, E.; Ates, H.; Yavasoglu, A.; Kirilmaz, L. A Novel Pilocarpine Microemulsion as an Ocular Delivery System: In Vitro and In Vivo Studies. *J. Clin. Exp. Ophthalmol.* **2015**, *06* (02), 1–6. https://doi.org/10.4172/2155-9570.1000408.

(44) Kumar, R.; Sinha, V. R. Preparation and Optimization of Voriconazole Microemulsion for Ocular Delivery. *Colloids Surfaces B Biointerfaces* **2014**, *117*, 82–88. https://doi.org/10.1016/j.colsurfb.2014.02.007.

(45) Ligório Fialho, S.; da Silva-Cunha, A. New Vehicle Based on a Microemulsion for Topical Ocular Administration of Dexamethasone. *Clin. Exp. Ophthalmol.* **2004**, *32* (6), 626–632. https://doi.org/10.1111/j.1442-9071.2004.00914.x.

(46) Abdul Rasool, B. K.; Salmo, H. M. Development and Clinical Evaluation of Clotrimazole-β-Cyclodextrin Eyedrops for the Treatment of Fungal Keratitis. *AAPS PharmSciTech* **2012**, *13* (3), 883–889. https://doi.org/10.1208/s12249-012-9813-4.
